# Cognitive Performance Under AI Advice: Development and Initial Validation of a CHC-Informed Assessment for Organizational Decision-Making

**DOI:** 10.3390/jintelligence14090223

**Published:** 2026-09-16

**Authors:** Filiz Mizrak, Turhan Karakaya, Burcak Vatansever Durmaz

**Affiliations:** 1 Management Information Systems, Atlas University, Istanbul 34408, Turkey; 2 Industrial Engineering, Dogus University, Istanbul 34680, Turkey; 3 Business Administration, Bahcesehir University, Istanbul 34350, Turkey; burcak.vatansever@bau.edu.tr

**Keywords:** artificial intelligence, Cattell–Horn–Carroll theory, cognitive performance, human–AI decision-making, performance-based assessment

## Abstract

Artificial intelligence (AI) is increasingly embedded in organizational decision-making, requiring employees not only to use AI-generated recommendations but also to evaluate their quality and determine when reliance is appropriate. Although established research examines behavioral reliance on algorithmic and AI advice, fewer studies have approached performance under AI advice as an individual-differences assessment problem integrating psychometric structure, cognitive correlates, process indicators, and criterion-related evidence. This study developed and initially validated a CHC-informed, performance-based assessment of cognitive performance under AI advice using 24 organizational decision scenarios. The assessment was designed around three closely related content/performance dimensions—AI error detection, evidence integration, and cognitive control and adaptive reliance—while also capturing confidence, response time, and reliance behavior. The validation sample comprised 780 employed adults in Türkiye. Psychometric analyses included confirmatory factor analysis, multidimensional item response theory, response-time analyses, scenario-level logistic regression, measurement invariance, differential item functioning, and internal cross-validation. Results indicated a dominant general cognitive-performance component together with additional structure corresponding to the three theoretically specified dimensions. Assessment performance was positively associated with established cognitive measures, including ICAR-16 reasoning performance, working memory, processing speed, and attentional control, whereas associations with AI-related self-reports were generally weaker. Dimension-aligned analyses supported the expected associations of ICAR-16 with AI error detection and working memory with evidence integration. Performance was also moderately associated with concurrently assessed organizational decision quality (r = 0.408) and explained additional variance in this criterion beyond demographic and work characteristics, AI experience, conventional cognitive-performance measures, and AI-related self-reports (Δ*R*^2^ = 0.103, *p* < .001). In contrast, several hypothesized scenario-specific associations involving AI confidence, time pressure, interruptions, and resistance to confidently inaccurate advice were not supported. Overall, the findings provide initial evidence for a performance-based approach to assessing how employees evaluate and respond to AI advice, while indicating that the proposed scenario-specific mechanisms and group-comparability findings require further replication before consequential applications are considered.

## 1. Introduction

Artificial intelligence is increasingly embedded in routine organizational decision-making rather than confined to specialist technical functions. Employees now encounter AI-generated recommendations, forecasts, rankings, summaries, and explanations across logistics, procurement, operations, marketing, human resources, finance, and risk management. In many of these settings, AI does not make the final decision autonomously; instead, employees must interpret machine-generated advice and decide whether to accept, modify, verify, or reject it. Recent work on human–AI collaboration therefore increasingly emphasizes the quality of joint decision-making rather than technology adoption alone (Do Khac & Leyer, 2026; Gonzalez & Heidari, 2025; Latto et al., 2026; Li & Tian, 2026).

This shift creates a practical and psychological challenge. AI-generated advice can be fluent, confident, and professionally presented even when it is inaccurate, incomplete, or based on weak assumptions. Hallucinations and reliability failures can therefore create risks when users infer correctness from presentation quality or apparent certainty (Cheng et al., 2026). At the same time, people do not respond to AI advice only on the basis of objective quality. Reliance is also shaped by trust, confidence, source information, and prior beliefs about the system (Ding et al., 2026; Hao et al., 2026; Meincke et al., 2026; Pearson et al., 2026). The quality of the AI output and the quality of the human response to that output are therefore distinct components of AI-supported decision performance.

A central concern is whether reliance is appropriately calibrated. Automation-bias research shows that users may give excessive weight to automated recommendations despite contradictory evidence, whereas algorithm-aversion research shows that users may also reject useful algorithmic advice after observing errors (Dietvorst et al., 2015; Lee & See, 2004; Romeo & Conti, 2026). More recent work similarly emphasizes that appropriate reliance depends on aligning trust and acceptance with the actual quality of the recommendation rather than maximizing trust in AI (Dogru & Krämer, 2025; Liebherr et al., 2026). Cognitive offloading adds another layer: AI can reduce effort, but reliance may become problematic when users disengage from information that still requires independent judgment (Gerlich, 2025; Zhu et al., 2026). These risks are especially relevant under time pressure, interruptions, competing information, and salient confidence cues.

Existing measures provide important but incomplete perspectives on this problem. AI-literacy scales assess knowledge, competencies, and beliefs about artificial intelligence (Carolus et al., 2023; Koch et al., 2024; Ng et al., 2024), AI self-efficacy measures assess perceived capability to use AI effectively (Morales-García et al., 2024; Wang & Chuang, 2024), and trust measures assess users’ evaluations of automated systems (Jian et al., 2000; Lee & See, 2004). These constructs are valuable, but they do not directly establish whether a person can identify an inaccurate recommendation, integrate conflicting evidence, revise an initial judgment, or resist a confidently expressed but unsupported conclusion in a specific decision. Performance-based human–AI research already examines advice taking, reliance, confidence, and decision accuracy; the measurement gap addressed here is therefore not the absence of behavioral assessment itself. Rather, it concerns the limited integration of repeated workplace decision scenarios into a psychometric individual-differences framework that can examine structure, cognitive correlates, process indicators, and criterion-related evidence within the same assessment.

Traditional cognitive measures address a different part of this gap. Tests of reasoning, working memory, processing speed, and attention provide established indicators of cognitive functioning, but they generally do not place these abilities inside AI-supported organizational decisions. The Cattell–Horn–Carroll (CHC) framework offers a useful organizing basis because it conceptualizes cognition as a hierarchy of broad and narrower abilities that contribute differently across tasks (Carroll, 1993; McGrew, 2009; Schneider & McGrew, 2018). In the present study, CHC is used as an interpretive framework rather than as the basis for proposing a new broad ability. Fluid reasoning may support evaluation of novel or inconsistent recommendations; working memory may support the maintenance and comparison of multiple constraints; processing speed may contribute to efficient evaluation under time limits; and attentional control may help maintain focus on diagnostic information when distracting or misleading cues are present.

Accordingly, the study conceptualizes cognitive performance under AI advice as an applied performance domain in which established cognitive resources are expressed while individuals evaluate machine-generated recommendations. The assessment was designed around three theoretically specified and closely related content/performance dimensions—AI error detection, evidence integration, and cognitive control and adaptive reliance—while confidence, response time, and reliance behavior were treated as complementary process indicators. This framing does not assume that the three dimensions are independent abilities or that working with AI constitutes a new form of intelligence. Instead, it treats them as related manifestations of performance within a common AI-supported decision context.

The present study therefore develops and initially validates a CHC-informed, performance-based assessment of cognitive performance under AI advice in organizational decision-making. Its contribution is primarily psychometric and integrative. First, it organizes repeated AI-supported workplace scenarios within an individual-differences assessment framework. Second, it examines whether performance shows a coherent psychometric structure while remaining related to established cognitive measures. Third, it combines final accuracy with confidence, response time, reliance behavior, and decision revision to characterize performance more fully than a total score alone. Fourth, it evaluates concurrent criterion-related and incremental validity beyond demographic and work characteristics, AI experience, conventional cognitive-performance measures, and AI-related self-reports. Finally, it examines preliminary group comparability through measurement-invariance and differential-item-functioning analyses. Scenario characteristics in the 24-item fractional design were fixed properties of particular scenarios rather than independently randomized manipulations; consequently, scenario-level coefficients are interpreted as adjusted associations and not as isolated causal effects.

## 2. Literature Review and Theoretical Framework

### 2.1. CHC-Informed Cognitive Foundations

The Cattell–Horn–Carroll (CHC) framework provides a useful structure for organizing the cognitive resources relevant to decision-making under AI advice. CHC theory conceptualizes cognitive functioning as a hierarchy of broad and narrower abilities that contribute differently across tasks (Carroll, 1993; McGrew, 2009; Schneider & McGrew, 2018). The present study applies this framework rather than proposing a new CHC ability. Cognitive performance under AI advice is treated as an applied performance domain in which established reasoning, memory, and processing resources are expressed while employees evaluate machine-generated recommendations. Fluid reasoning, working memory, and processing speed provide the principal CHC-informed cognitive foundations, while attentional control is treated as a complementary individual-difference resource relevant to maintaining task goals in the presence of distracting or misleading information.

Fluid reasoning is relevant when employees must evaluate unfamiliar problems, detect inconsistencies, compare alternative explanations, or determine whether a recommendation follows from the available evidence (Carroll, 1993; McGrew, 2009). Measures such as the International Cognitive Ability Resource (ICAR) provide established performance-based indicators of reasoning-related individual differences (Condon & Revelle, 2014), while cognitive-reflection research shows that individuals differ in their tendency to reconsider an initially attractive response (Frederick, 2005; Thomson & Oppenheimer, 2016). These capabilities are relevant when AI advice is fluent and plausible but contains an unsupported inference, omission, or contradiction (Corvelo Benz & Gomez Rodriguez, 2025; Gonzalez & Heidari, 2025).

Working memory supports the temporary maintenance and manipulation of information while other cognitive operations are performed (Conway et al., 2005; Unsworth et al., 2005). In AI-supported organizational decisions, employees may need to retain policy requirements, numerical evidence, stakeholder constraints, and elements of an AI recommendation while comparing their implications. This function is especially relevant to evidence integration. AI can reduce cognitive burden through offloading, but offloading is not uniformly beneficial when it substitutes for independent evaluation rather than supporting it (Gerlich, 2025; Zhu et al., 2026).

Processing speed contributes to the efficient evaluation of relevant information when decisions must be made within limited time. Faster responding, however, is not necessarily better: rapid responses may reflect either efficient processing or premature acceptance of AI advice. Response time is therefore interpreted jointly with accuracy rather than as an isolated indicator of ability, consistent with psychometric approaches that examine accuracy and speed together (Guo et al., 2022; Zhang et al., 2026).

Attentional control is conceptually relevant because AI-supported decisions may contain interruptions, salient confidence cues, and competing information. Research on selective and executive attention shows that individuals differ in their ability to maintain task goals while resisting distraction (Eriksen & Eriksen, 1974; Fan et al., 2002). In the present framework, attentional control is not treated as a newly established CHC dimension of the assessment. Instead, it is a companion individual-difference resource expected to support sustained focus, resistance to misleading cues, and recovery from distraction (Galindo-Domínguez et al., 2026).

These cognitive resources are expected to overlap in realistic decisions rather than map exclusively onto one observable behavior. The framework therefore uses CHC as an organizing foundation while allowing substantial shared variance across performance tasks. Comprehension–knowledge may also contribute to scenario understanding, but specialized occupational terminology was minimized and the information needed to solve each problem was provided within the scenarios to reduce dependence on job-specific knowledge.

**Figure 1:**
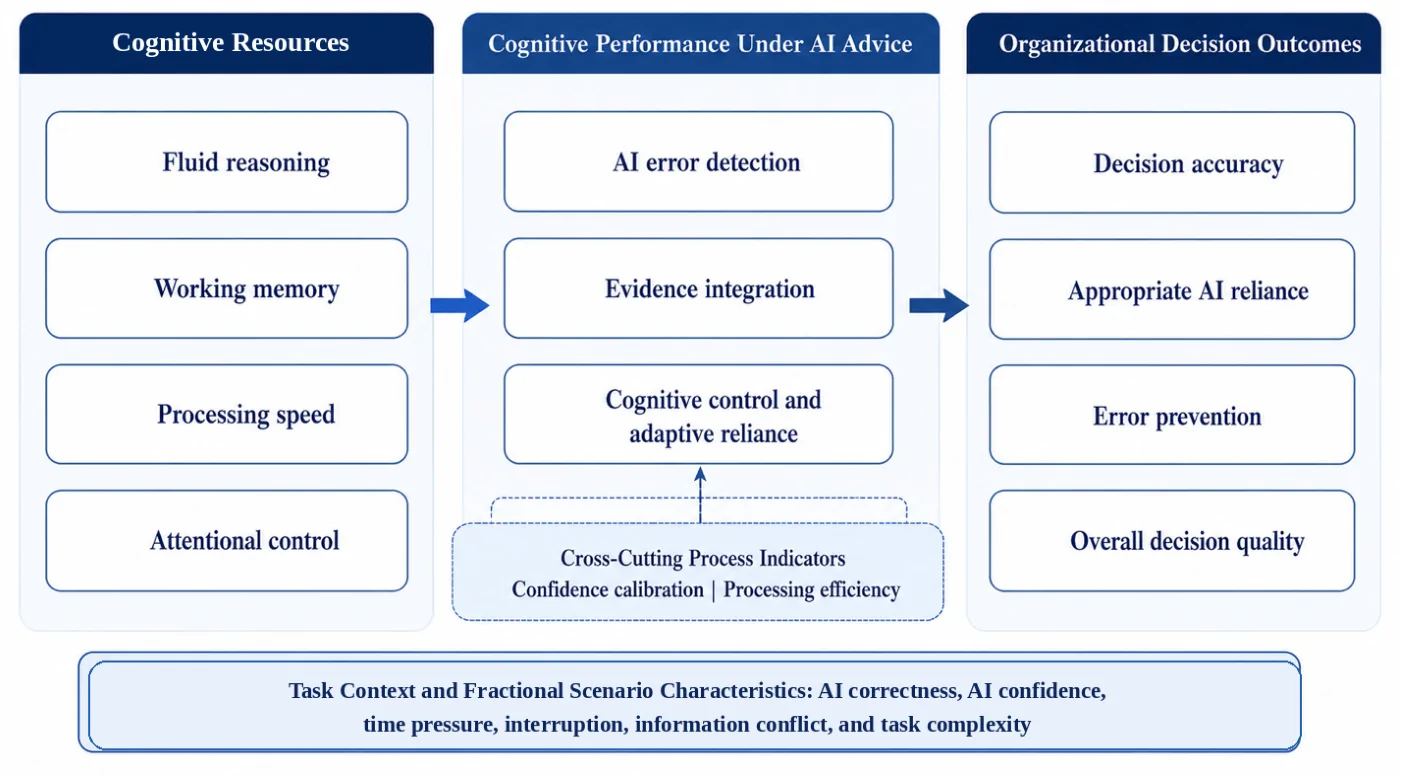
Conceptual Framework of Cognitive Performance Under AI Advice. Note. The three content/performance dimensions are closely related and share a substantial general cognitive-performance component; the arrows represent theoretical relationships rather than independent cognitive abilities.

Figure 1 summarizes the CHC-informed framework, showing how established cognitive resources and attentional control may support performance when employees evaluate AI advice. The arrows represent theoretical relationships to be examined rather than evidence that the proposed performance dimensions are independent cognitive abilities.

### 2.2. Human–AI Decision-Making and Appropriate Reliance

AI-generated recommendations are increasingly incorporated into organizational decisions involving logistics, procurement, customer service, finance, human resources, operations, forecasting, and risk assessment. In these settings, AI may rank alternatives, generate forecasts, flag anomalies, summarize evidence, or recommend actions, but a human decision-maker often remains responsible for interpreting and acting on that output. Research on human–AI collaboration therefore emphasizes complementarity between machine-generated information and human judgment rather than simple substitution (Do Khac & Leyer, 2026; Gonzalez & Heidari, 2025; Latto et al., 2026; Li & Tian, 2026).

A central concept in this literature is appropriate reliance. Trust is useful when it is calibrated to actual system capability rather than maximized indiscriminately (Lee & See, 2004). Contemporary studies likewise show that expertise, self-confidence, advice quality, and perceived trustworthiness shape whether users accept or reject AI recommendations (Ding et al., 2026; Dogru & Krämer, 2025; Liebherr et al., 2026). The relevant behavioral criterion is therefore not high trust itself but whether reliance changes appropriately with recommendation quality.

Reliance can fail in both directions. Overreliance occurs when faulty or insufficiently supported AI advice is accepted despite contradictory evidence, whereas underreliance occurs when useful advice is rejected without adequate reason. Automation bias and hallucinated but credible-looking outputs can increase the risk of inappropriate acceptance (Cheng et al., 2026; Pearson et al., 2026; Romeo & Conti, 2026), while algorithm-aversion research shows that users may also reject algorithmic advice after observing errors even when the system remains useful overall (Dietvorst et al., 2015). Effective performance therefore requires discriminating between advice that should be accepted, questioned, verified, or rejected.

Confidence cues further complicate this judgment because fluent or confidently expressed recommendations may be treated as more credible even when confidence is not well calibrated to correctness. Advice quality, system confidence, and users’ own confidence can influence acceptance behavior (Dogru & Krämer, 2025; Hao et al., 2026; Meincke et al., 2026). Time pressure, interruptions, and information conflict may also alter the opportunity to evaluate evidence. These factors motivate the scenario-level hypotheses examined in this study.

Importantly, behavioral research on automation, advice taking, and human–AI reliance already evaluates actual choices and acceptance behavior; the present study does not claim that behavioral measurement of AI reliance is new (Corvelo Benz & Gomez Rodriguez, 2025; Mayer et al., 2026; Pearson et al., 2026). The intended contribution is psychometric and integrative: repeated organizational scenarios are used to examine whether individual differences in performance under AI advice can be organized into a coherent assessment framework and related systematically to established cognitive measures, AI-related self-reports, process indicators, and an independent concurrent decision-quality criterion.

The scenario characteristics used to examine these relationships were fixed properties of particular scenarios in a fractional design rather than independently crossed manipulations within otherwise identical content. Accordingly, theoretical expectations concerning AI correctness, expressed confidence, time pressure, interruptions, information conflict, and task complexity are evaluated as adjusted associations within the observed scenario set, not as isolated causal effects.

This distinction is important for the present framework: the assessment is intended primarily to characterize performance and its correlates, while the scenario-level conditions provide secondary tests of theoretically motivated contextual associations.

### 2.3. Measurement Gap and Assessment Framework

Existing AI-related instruments provide valuable information about what people know, believe, and feel capable of doing. AI-literacy measures assess knowledge and competencies (Carolus et al., 2023; Koch et al., 2024; Ng et al., 2024), AI self-efficacy measures assess perceived capability (Morales-García et al., 2024; Wang & Chuang, 2024), and trust measures assess evaluations of automated systems (Jian et al., 2000; Lee & See, 2004). These constructs are relevant to AI-supported work, but they are not interchangeable with demonstrated performance in a specific decision. A person may understand that AI can make errors, for example, without detecting an error when evaluating a concrete recommendation.

Conventional cognitive assessments address a complementary question. Measures of reasoning, cognitive reflection, working memory, processing speed, and attention capture cognitive resources associated with effective judgment (Condon & Revelle, 2014; Conway et al., 2005; Fan et al., 2002; Frederick, 2005; Unsworth et al., 2005), but they were not designed to reproduce the specific demands of evaluating AI advice together with organizational evidence, confidence cues, and competing constraints. The measurement gap is therefore not the absence of behavioral or cognitive research; it is the limited integration of these traditions within a psychometric assessment of repeated AI-supported workplace decisions.

Cognitive performance under AI advice is defined here as an individual’s capacity to evaluate, verify, integrate, and appropriately act on AI-generated information while making organizational decisions. The assessment was theoretically organized around three closely related content/performance dimensions: AI error detection, evidence integration, and cognitive control and adaptive reliance. These dimensions describe recurring task demands rather than three presumed independent abilities. Confidence calibration and processing efficiency are treated as cross-cutting process indicators.

AI error detection concerns recognizing factual inaccuracies, unsupported claims, logical inconsistencies, important omissions, or contradictions between an AI recommendation and the evidence provided. Fluid reasoning is expected to be particularly relevant because users must determine whether a conclusion follows from the available information rather than judge it by surface plausibility alone (Cheng et al., 2026; Romeo & Conti, 2026).

Evidence integration concerns combining AI advice with numerical information, policies, contextual evidence, stakeholder requirements, and competing operational constraints. Working memory is expected to be especially relevant because several pieces of information may need to remain active while their implications are compared (Conway et al., 2005; Unsworth et al., 2005). This dimension reflects integration of machine-generated information with task evidence rather than simple agreement with the AI recommendation.

Cognitive control and adaptive reliance concerns regulating the influence of AI advice on the final judgment. Relevant behaviors include resisting automatic acceptance, reconsidering an initial decision, overriding inaccurate advice when evidence warrants it, and retaining useful advice when it is supported. Strong performance therefore does not imply rejecting AI more often; it implies differentiating between recommendations that merit acceptance and those that require challenge or verification (Dogru & Krämer, 2025; Liebherr et al., 2026; Pearson et al., 2026).

Confidence calibration and processing efficiency provide information across all three content/performance dimensions. Calibration concerns the correspondence between subjective confidence and actual correctness, whereas processing efficiency concerns response time interpreted jointly with response quality. The assessment therefore records final accuracy, evaluation of AI advice, confidence, acceptance or rejection of advice, decision revision, and response time rather than treating final correctness as the only informative outcome (Guo et al., 2022; Zhang et al., 2026).

The proposed mappings are theoretical emphases rather than exclusive one-to-one correspondences. Fluid reasoning may contribute to evidence integration, working memory may support error detection, and all three content/performance dimensions may share substantial general performance variance. The framework therefore predicts related dimensions and leaves open whether a dominant general cognitive-performance component accounts for much of their common variance. This issue is evaluated empirically by comparing alternative measurement models rather than assumed in advance.

Table 1 summarizes the cognitive foundations, proposed content/performance dimensions and cross-cutting process indicators, observable task behaviors, and scoring indicators used in the framework.

### 2.4. Research Questions and Hypotheses

The framework leads to six research questions addressing structure, measurement precision, processing efficiency, validity, incremental concurrent validity, and preliminary group comparability:

RQ1: What dimensional structure best represents cognitive performance under AI advice?

RQ2: How effectively do the assessment tasks distinguish employees with different levels of performance?

RQ3: What is the relationship between response accuracy and processing speed?

RQ4: Does assessment performance show convergent and discriminant relationships with established cognitive measures and AI-related self-reports?

RQ5: Does assessment performance explain incremental concurrent variance in organizational decision quality beyond demographic and work characteristics, AI experience, conventional cognitive-performance measures, and AI-related self-reports?

RQ6: Does the assessment show broadly comparable measurement functioning across the demographic, occupational, managerial, and AI-use groups examined?

The directional hypotheses concern associations observed within the fractional scenario design. Because scenario characteristics were fixed to particular scenarios rather than independently randomized within otherwise identical content, these hypotheses should not be interpreted as tests of isolated causal effects:

**H1.**  *Among scenarios containing incorrect AI advice, final decision accuracy will be lower when the advice is expressed with high rather than low confidence.*

**H2.**  *Inappropriate acceptance of incorrect AI recommendations will be higher in time-pressure scenarios than in scenarios without time pressure.*

**H3.**  *Decision accuracy will be lower in scenarios containing digital interruptions, particularly when evidence integration is central to the task.*

**H4.**  *Fluid reasoning and working memory will be positively associated with AI error-detection and evidence-integration performance, respectively.*

**H5.**  *Higher overall cognitive performance under AI advice will be associated with lower susceptibility to confidently expressed inaccurate AI recommendations.*

**H6.**  *Assessment performance will explain incremental concurrent variance in organizational decision quality beyond demographic and work characteristics, AI experience, conventional cognitive-performance measures, and AI-related self-reports.*

## 3. Materials and Methods

### 3.1. Assessment Development

The assessment was developed through construct specification, scenario design, expert review, cognitive interviewing, pilot testing, and item refinement. The conceptual framework was CHC-informed rather than intended to define a new CHC ability. Fluid reasoning, working memory, and processing speed were treated as established cognitive resources, while attentional control was treated as a companion individual-difference resource. The 24 scenarios were theoretically organized into three closely related content/performance dimensions—AI error detection, evidence integration, and cognitive control and adaptive reliance—with confidence calibration and processing efficiency treated as cross-cutting process indicators.

An initial pool of 30 organizational scenarios was developed across broadly applicable contexts, including supplier selection, logistics disruption, inventory planning, employee scheduling, project prioritization, customer complaints, budget allocation, risk evaluation, marketing, recruitment, quality control, and operational planning. Specialized occupational terminology was minimized to reduce dependence on job-specific knowledge. Each scenario presented a concise organizational problem, task-relevant evidence, an AI-generated recommendation, and a required decision. Participants made an initial judgment, evaluated the AI advice, made a final decision, reported confidence, and could retain or revise their original response. Response-time variables were derived from the timing information recorded for the pre- and post-advice response stages.

The complete 24-scenario assessment administered in the study, including scenario evidence, AI-generated recommendations, response options, scoring keys, and scenario-characteristic coding, is provided in Supplementary S2 of the Supporting Information. The complete scenario-level blueprint is reported in Table S1.

Table 2 summarizes the assessment blueprint by linking each content/performance dimension to its organizational task context, principal cognitive demand, relevant scenario characteristics, required response, and process indicator.

Scenario presentation order was randomized, but the scenario characteristics themselves were fixed properties of particular scenarios in a fractional design rather than independently randomized within otherwise identical content. The final assessment contained 12 scenarios with correct and 12 with incorrect AI advice; 12 high- and 12 low-confidence AI cues; 6 scenarios with time pressure and 18 without; 4 scenarios with an interruption and 20 without; 12 high- and 12 low-information-conflict scenarios; and 8 low-, 8 moderate-, and 8 high-complexity scenarios. Consequently, coefficients for these characteristics are interpreted as adjusted associations within the observed scenario set rather than as isolated causal effects. Table 3 summarizes the scenario characteristics embedded in the final assessment and the principal cognitive demands and outcomes associated with each characteristic.

The original 30 scenarios were reviewed by 10 experts representing psychometrics, cognitive assessment, supply chain decision-making, human–AI interaction, and organizational decision-making. Their professional experience averaged 15.9 years. Experts evaluated relevance, clarity, realism, cognitive demand, occupational neutrality, and ambiguity. Across the initial pool, the scale-level average CVI was 0.800. Twenty-four scenarios achieved I-CVI values of at least 0.80; within this group, I-CVI values ranged from 0.80 to 1.00, CVRs from 0.60 to 1.00, and modified kappa values from approximately 0.79 to 1.00. Their S-CVI/Ave was 0.908. Complete item-level results and the expert evaluation form are provided in Table S2 and Supplementary S3.

Cognitive interviews were then conducted with 20 employed participants representing procurement, customer service, planning, operations, warehousing, and transport functions. Mean ratings were 4.40/5 for clarity, 4.20/5 for realism, and 3.05/5 for perceived difficulty. Eleven participants identified no substantive problems; the remaining interviews identified calculation burden, ambiguous evidence, instruction wording, or terminology that required refinement. These findings were used to simplify unnecessary calculations and clarify wording without reducing the intended cognitive demands. Full cognitive-interview findings and the interview protocol are provided in Table S3 and Supplementary S4.

Pilot testing involved 120 employees. Across the 30 candidate scenarios, mean difficulty was 0.512, mean discrimination was 0.376, and mean response time was 47.53 s. Twenty-three scenarios were retained without major change. Scenario I06 showed acceptable content validity but weaker discrimination and was refined rather than removed. Scenarios I25–I30 showed a less favorable combination of content validity, discrimination, response burden, and interpretability and were removed, producing the final 24-scenario assessment. Complete pilot statistics are reported in Table S4. Table 4 summarizes the content-validation and pilot evidence used in the item-selection decisions.

**Figure 2:**
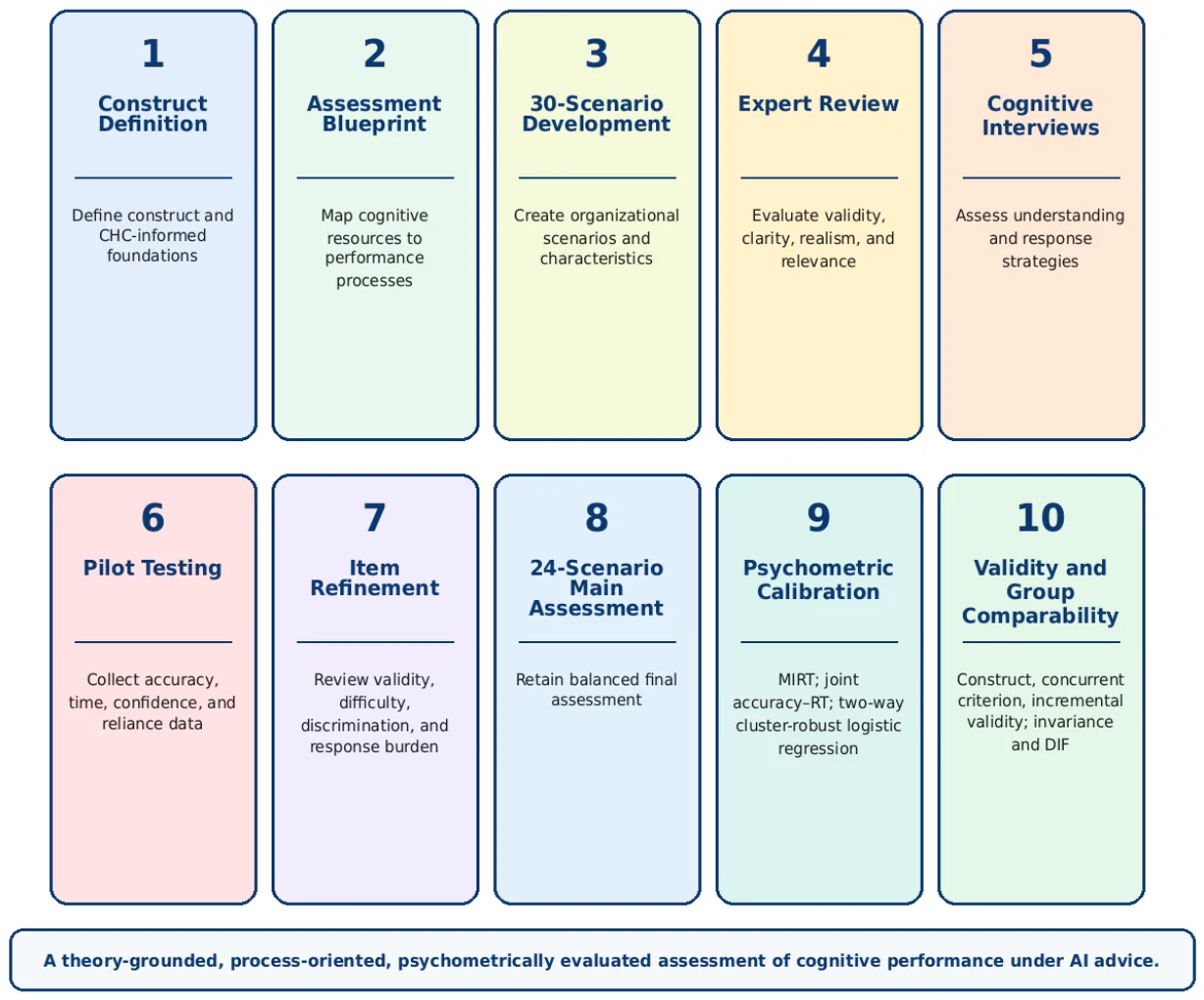
Assessment Development and Initial Validation Process.

Item-retention decisions considered content validity, cognitive-interview evidence, item difficulty, discrimination, response burden, ambiguity, and occupational neutrality jointly rather than relying on a single statistical threshold. Figure 2 summarizes the resulting development sequence from construct definition through psychometric evaluation and validity testing.

### 3.2. Participants and Procedure

Data were collected in Türkiye from 13 May to 10 June 2026 using a nonprobability purposive employee-recruitment strategy. Potential participants were reached through company email invitations and LinkedIn. The study was administered online through Google Forms in Turkish, and no financial or other participation compensation was provided. Eligibility required participants to be at least 18 years old, currently employed at least part-time, use digital technologies at work, and have basic familiarity with AI-supported tools or recommendations; advanced AI expertise was not required.

The dataset initially contained 900 participant records; 780 were retained after the prespecified eligibility and data-quality screening procedures described below. The final sample ranged from 20 to 64 years of age (*M* = 38.82, *SD* = 9.31) and averaged 10.50 years of work experience (*SD* = 5.29). Participants represented transport operations, procurement, warehousing, inventory planning, customer operations, supply chain analytics, and management. Detailed demographic, occupational, and AI-use characteristics are reported in Table 5. No single-effect a priori power analysis was used because the primary aim was psychometric development and validation across multiple models rather than testing one focal effect. The final *N* = 780 yielded 18,720 participant-by-scenario observations and substantial subgroup sizes for the planned psychometric and group-comparability analyses, while inference involving scenario clustering was treated conservatively because only 24 scenarios were available.

Participants first completed demographic, occupational, and AI-experience questions, followed by the 24-scenario organizational AI-advice assessment. They then completed the companion cognitive-performance measures, AI-related self-report measures, and the organizational decision-quality criterion task. Scenario presentation order was randomized; the scenario characteristics described in Table 3 were not independently randomized within scenario content. The administration captured initial and final decisions, AI-evaluation responses, confidence, acceptance or rejection of AI advice, decision revision, and the response-time variables available for the pre- and post-advice stages. Full focal assessment instructions are provided in Supplementary S1. Table 5 illustrates the characteristics of the final analytic sample.

Participation was voluntary and anonymous, and informed consent was obtained before data collection. Ethical approval was granted before recruitment by the Istanbul Atlas University Social and Humanities Research and Publication Ethics Committee on 13 April 2026 (Decision No. 03/02; Official Document No. E-22686390-050.99-95101). Participants could discontinue participation at any time.

### 3.3. Measures and Task Design

The focal measure was the 24-scenario performance-based organizational AI-advice assessment developed in Section 3.1. Each scenario presented an organizational problem and relevant evidence followed by an AI-generated recommendation. Participants made an initial judgment, evaluated the AI advice, made a final decision, and rated confidence on a 0–10 scale. The principal 24-scenario indicators were final decision accuracy (0–24), AI-evaluation accuracy (0–24), appropriate reliance (0–24), confidence-calibration indices, response-time measures, and decision revision. AI-evaluation accuracy refers to the correct evaluation of the AI recommendation across all 24 scenarios and is distinct from the eight-scenario AI-error-detection content/performance dimension. The three dimension scores were based on the eight scenarios assigned to each theoretically specified content/performance grouping.

Supplementary S6 and Table S14 document the study-specific variables, response coding, and scoring rules. Published measures are identified by their original sources. All study materials were administered in Turkish, and scenario wording and task comprehensibility were evaluated through the cognitive-interview and pilot stages described above. The present study does not claim a separate translation-validation study for the published measures.

Conventional cognitive-performance measures were included to examine convergent relationships. Fluid/general reasoning was measured with the 16-item International Cognitive Ability Resource (ICAR-16) (Condon & Revelle, 2014; Dworak et al., 2021), scored 0–16 (α = 0.809). Cognitive reflection was assessed with four binary-scored items based on established cognitive-reflection tasks (Frederick, 2005; Thomson & Oppenheimer, 2016), scored 0–4 (α = 0.542). Working memory was represented by a brief aggregate performance score informed by working-memory span principles (Conway et al., 2005; Unsworth et al., 2005); observed scores ranged from 3 to 14. Processing speed was represented by a brief timed performance score; observed scores ranged from 30 to 80. The retained dataset contains aggregate working-memory and processing-speed scores rather than trial-level responses, so item-level internal-consistency estimates are not reported for these two companion measures. Attentional control was represented by eight study-specific dichotomously scored indicators (ATT1–ATT8), coded 0/1 and summed to a 0–8 total (α = 0.718). Because these indicators were study-specific rather than a published standardized attention task, attentional control was treated as a companion individual-difference measure rather than as a separately validated CHC ability in the focal assessment.

AI-related measures included MAILS-10 (Carolus et al., 2023; Koch et al., 2024), scored as the mean of 10 six-point items (α = 0.914), and a 12-item trust-in-AI measure (Jian et al., 2000), scored on a six-point scale (α = 0.921). AI self-efficacy was measured with six items scored from 1 to 6 and averaged across items (α = 0.873). AI-use frequency was coded ordinally from 1 (rarely) to 5 (several times daily). Prior generative-AI experience was recorded as Limited, Moderate, or Extensive and coded 1, 2, and 3, respectively. These variables were used as related self-report constructs or experience controls rather than as substitutes for demonstrated performance.

Concurrent criterion-related validity was evaluated with a separately scored six-item organizational decision-quality task. Each item was scored correct or incorrect and summed from 0 to 6 (α = 0.748). Because the criterion task and focal assessment were administered in the same study session, the resulting evidence is interpreted as concurrent rather than predictive validity. The criterion was scored independently from the focal 24-scenario assessment. Table 6 shows the measures, scoring procedures, reliability information, and analytic roles.

### 3.4. Data Analysis

Analyses proceeded from data screening and item diagnostics to dimensionality, item-response modelling, process analysis, validity testing, and preliminary group-comparability assessment. Analyses were implemented in R version 4.6.1 and Python version 3.14.7; the complete software, package, version, coding, and reproducibility specification is provided in Supplementary S5 of the Supporting Information. Screening considered missingness, incomplete assessments, technical failures, repeated response patterns, implausibly short completion times, and extreme response-time observations. Very slow responses were not removed solely because of their duration because long response times may reflect genuine processing difficulty.

Preliminary item analysis examined difficulty, discrimination, corrected item–total correlations, response-time intensity, confidence distributions, and potential floor or ceiling effects. For binary performance items, difficulty was the proportion correct. Dimensionality was evaluated using tetrachoric correlations, parallel analysis, and the minimum average partial procedure. Confirmatory models compared one-factor, correlated three-factor, higher-order, and bifactor specifications. CFI, TLI, RMSEA, SRMR, AIC, and BIC were considered jointly, and the correlated dimensions were not assumed to represent independent abilities.

Item-level measurement properties were evaluated with two-parameter logistic (2PL) item-response models. With *i* denoting participants and *j* denoting items, the probability of a correct response was represented as follows:

Item-level measurement properties were then examined using two-parameter logistic item response theory models. For participant j responding to item i, the probability of a correct response was represented as

(1) $$P\left(X_{ij}=1|\theta_{i}\right)=\frac{1}{1+exp\left[-a_{j}\left(\theta_{i}-b_{j}\right)\right]}$$

Here, *a_j_* denotes item discrimination, *b_j_* item difficulty, and *θ_i_* the participant’s latent performance level. A unidimensional 2PL model was compared with a correlated multidimensional 2PL model and a bifactor specification. Item fit, information, conditional standard errors, and model fit were examined. Conditional measurement precision was summarized as:

(2) $$SE\left(\theta \right)=\frac{1}{\sqrt{I\left(\theta \right)}}$$

where *I*(*θ*) is the test information available at a given latent-performance level.

Accuracy and response time were also examined jointly using a log-normal response-time component. With *i* denoting participants and *j* scenarios, the timing component was represented as:

(3) $$log\left(T_{ij}\right)=\lambda_{j}-\nu_{i}+\varepsilon_{ij},$$

where *T_ij_* is response time, *λ_j_* is scenario time intensity, *ν_i_* is participant processing speed, and *ε_ij_* is the residual term. Response time was interpreted jointly with accuracy; faster responding was not assumed to indicate better performance by itself.

The primary scenario-level analysis modeled binary final decision accuracy using logistic regression with two-way participant- and scenario-clustered covariance estimation. The prespecified primary model included AI-advice correctness, AI confidence, time pressure, interruption, information conflict, a high-complexity contrast, ICAR-16, working memory, processing speed, attentional control, AI literacy, correctness × confidence, correctness × time pressure, and interruption × working memory. Continuous person-level predictors were standardized before estimation. The model estimated an ordinary logistic mean structure; participant and scenario random intercepts were not included in the primary model.

(4) $$logit\left[P\left(Y_{ij}=1\right)\right]=\beta_{0}+x_{j}^{\prime }\beta +z_{i}^{\prime }\gamma +w_{ij}^{\prime }\delta$$

In Equation (4), *x_j_* denotes the vector of scenario characteristics, *z_i_* the standardized person-level predictors, and *w_ij_* the prespecified interaction terms. Because the smaller clustering dimension contained only 24 scenarios, cluster-robust inference was reported conservatively using a t distribution with 23 degrees of freedom. Robustness was also examined through leave-one-scenario-out refits and an alternative scenario-random-intercept model. A fully crossed participant- and scenario-random-intercept model was explored but was not used for inference because it did not converge reliably. The previously considered remaining-23-scenario performance score was not included in the primary model because it is a within-assessment rest score rather than an independent cognitive measure; models using the rest score, balanced split-half scores, other-dimension scores, and an independent cognitive composite are reported as sensitivity analyses in the Supporting Information. H5 was evaluated directly among incorrect-AI scenarios by testing whether participant performance moderated the association between high AI confidence and decision accuracy/reliance.

Confidence ratings were rescaled from 0–10 to 0–1 and evaluated with calibration bias, absolute calibration error, and the Brier score (Brier, 1950). Calibration bias was calculated as:

(5) $$\mathrm{Calibration}\;\mathrm{Bias}=\frac{1}{N}\sum_{k}\left(c_{k}-y_{k}\right),$$

where *c_k_* is reported confidence and *y_k_* is observed correctness coded 0 or 1. Positive values indicate overconfidence and negative values underconfidence. Absolute calibration error was calculated as:

(6) $$ACE=\frac{1}{N}\sum_{k}\left|c_{k}-y_{k}\right|,$$

and probabilistic calibration was additionally summarized using the Brier score:

(7) $$\mathrm{Brier}\;\mathrm{Score}=\frac{1}{N}\sum_{k}{\left(c_{k}-y_{k}\right)}^{2}$$

Lower absolute calibration error and Brier scores indicate better calibration. Confidence discrimination was examined by comparing confidence for correct and incorrect decisions and by inspecting calibration patterns across relevant scenario characteristics.

Validity analyses distinguished convergent, discriminant, concurrent criterion-related, and incremental evidence. Convergent analyses examined relationships with ICAR-16, cognitive reflection, working memory, processing speed, and attentional control; H4 was additionally evaluated with dimension-aligned correlations linking ICAR-16 to AI error detection and working memory to evidence integration. AI literacy, trust in AI, AI self-efficacy, and AI experience were treated as related but conceptually distinct constructs. Incremental concurrent validity was evaluated hierarchically: Model 1 entered demographic variables; Model 2 added work experience; Model 3 added AI-use frequency and prior generative-AI experience; Model 4 added ICAR-16, cognitive reflection, working memory, and processing speed; Model 5 added AI literacy, trust in AI, and AI self-efficacy; and Model 6 added focal 24-scenario final accuracy. Attentional control was evaluated separately in a sensitivity model because its status differs from the conventional cognitive performance block. Ten-fold cross-validation was used as an internal assessment of statistical stability and is not interpreted as prospective external validation. Incremental contribution was summarized as:

(8) $$\Delta R^{2}=R_{new}^{2}-R_{previous}^{2}$$

Measurement invariance and differential item functioning (DIF) were examined as preliminary group-comparability tests. Two-group invariance comparisons used male (*n* = 427) versus female (*n* = 348), age <40 years (*n* = 415) versus ≥40 years (*n* = 365), bachelor’s degree or below (*n* = 498) versus postgraduate education (*n* = 282), nonmanager (*n* = 546) versus manager (*n* = 234), daily-or-more AI use (*n* = 433) versus weekly-or-less use (*n* = 347), and operations-oriented roles (*n* = 374) versus planning/analytical roles (*n* = 406). The five participants identifying as nonbinary/another gender were retained in overall analyses but were not included in the two-group gender invariance comparison. Invariance progressed from configural to loading and threshold models, with residual constraints examined where appropriate. Logistic DIF models used the remaining 23 items as the matching score and tested uniform group and non-uniform group-by-matching-score terms. Benjamini–Hochberg false-discovery-rate adjustment was applied separately to uniform- and non-uniform-DIF test families within each group comparison, and statistical significance was interpreted together with ΔMcFadden *R*^2^ and adjusted probability differences rather than as proof of fairness.

## 4. Results

### 4.1. Sample Characteristics, Data Quality, and Preliminary Item Results

The dataset contained 900 participant records. Following eligibility and data-quality screening, 780 cases were retained for analysis and 120 were excluded. Exclusions were due to implausibly short completion times (*n* = 33), incomplete assessments (*n* = 28), technical failures (*n* = 19), external AI assistance (*n* = 17), repeated response patterns (*n* = 15), or failure to meet the eligibility criteria (*n* = 8). Among the 780 retained cases, no missing values were observed in the demographic, scenario-level, or scale-item variables used in the analyses.

Participants ranged from 20 to 64 years of age (*M* = 38.82, *SD* = 9.31) and reported an average of 10.50 years of work experience (*SD* = 5.29). The sample included 427 men (54.7%), 348 women (44.6%), and 5 participants identifying as nonbinary or another gender (0.6%). Occupational representation included transport operations (20.9%), procurement (16.4%), warehousing (16.0%), inventory planning (15.5%), customer operations (11.0%), supply chain analytics (10.6%), and management (9.5%). AI use was relatively frequent: 15.1% used AI several times daily, 40.4% daily, 25.9% weekly, 11.7% monthly, and 6.9% rarely.

Response times were screened across the 18,720 participant–scenario observations. Raw pre-AI and post-AI response times showed moderate positive skewness of 0.77 and 0.78, respectively. Log transformation reduced the skewness to −0.04 and −0.01. Using an absolute standardized log-response-time threshold of 3.29, 19 pre-AI responses and 9 post-AI responses were flagged as extreme, representing less than 0.1% of observations. These responses were not automatically excluded because unusually slow responses could reflect genuine processing difficulty. Mean participant-level response time was 46.96 s before AI advice (*SD* = 5.13) and 35.84 s after AI advice (*SD* = 3.99).

Participants answered an average of 11.40 of the 24 scenarios correctly (*SD* = 5.66), corresponding to an overall accuracy rate of 0.475. Only one participant obtained a score of zero and seven achieved the maximum score of 24, indicating minimal floor and ceiling effects. The full 24-item accuracy score showed good internal consistency (α = 0.877). Reliability was also acceptable for the three content/performance groupings: α = 0.726 for AI error detection, α = 0.734 for evidence integration, and α = 0.778 for cognitive control and adaptive reliance. Correlations among these grouping scores ranged from 0.571 to 0.594, indicating substantial overlap alongside content-specific variation.

Table 7 summarizes the principal study variables. Final accuracy was positively related to AI-evaluation accuracy (r = 0.594), appropriate reliance (r = 0.646), ICAR-16 performance (r = 0.461), working memory (r = 0.343), cognitive reflection (r = 0.339), processing speed (r = 0.259), and attentional control (r = 0.440), all *p* < .001. Associations with AI literacy (r = 0.246) and AI self-efficacy (r = 0.161) were smaller, while trust in AI was essentially unrelated to performance (r = −0.007, *p* = .841). Frequency of AI use also showed only a weak relationship with final accuracy, Spearman’s ρ = 0.063, *p* = .077. Final accuracy was moderately associated with the concurrently administered organizational decision-quality criterion, r = 0.408, *p* < .001.

Preliminary item analysis indicated satisfactory variation across the 24 retained scenarios. Item difficulty, defined as the proportion of correct final decisions, ranged from 0.181 to 0.824 (*M* = 0.475). Upper–lower 27% discrimination indices ranged from 0.351 to 0.791 (*M* = 0.589), while corrected item–total correlations ranged from 0.301 to 0.566 (*M* = 0.446). Mean post-AI response times ranged from 26.86 to 46.70 s. S24 was the easiest scenario (*p* = 0.824), whereas S09 was the most difficult (*p* = 0.181). S22 showed the strongest discrimination (*D* = 0.791) and corrected item–total relationship (r = 0.566). Even the weakest retained item, S24, maintained a corrected item–total correlation above 0.30. Accordingly, all 24 scenarios were retained for the subsequent dimensionality and IRT analyses. Complete item-level descriptive statistics are provided in Table S5. Table 8 demonstrates the preliminary item-level performance of the 24 retained scenarios, including difficulty, discrimination, item-total relationships, response time, and retention decisions.

### 4.2. Dimensionality and Multidimensional IRT Results

Dimensionality was examined using the 24 binary final-decision items from the 780 retained cases. Parallel analysis of the tetrachoric correlation matrix revealed a strong first dimension. The first observed eigenvalue was 10.08, well above the corresponding 95th-percentile random eigenvalue of 1.66. The second observed eigenvalue was 1.55 and was essentially equal to, but slightly below, its random criterion of 1.55, while the third observed eigenvalue of 1.32 was below the corresponding random value of 1.51. Thus, parallel analysis primarily identified a strong general component, with only borderline evidence for a second component. In contrast, the minimum average partial procedure reached its minimum after three components, suggesting that meaningful multidimensional structure remained after accounting for the dominant general component.

Confirmatory model comparisons clarified this pattern. The one-factor model showed inadequate fit, χ^2^(252) = 2106.06, CFI = 0.799, TLI = 0.780, RMSEA = 0.097, and SRMR = 0.063. Fit improved substantially for the theoretically specified correlated three-factor model, χ^2^(249) = 1207.78, CFI = 0.896, TLI = 0.885, RMSEA = 0.070, and SRMR = 0.043. Standardized loadings were positive and ranged from 0.472 to 0.811. The three factors were strongly correlated (0.756–0.798), indicating that the content/performance dimensions were distinguishable in specification but shared considerable common variance. Because CFI and TLI remained below commonly preferred levels, the three-factor CFA is interpreted as an improvement over the one-factor model rather than as unequivocal evidence of three sharply distinct latent abilities.

A higher-order model produced essentially the same fit as the correlated three-factor model because only three first-order factors were specified. Higher-order loadings ranged from 0.863 to 0.911, indicating a substantial common cognitive-performance component. The bifactor model yielded CFI = 0.904, TLI = 0.883, RMSEA = 0.071, and SRMR = 0.043; approximately 74.7% of common loading variance was attributable to the general factor. Taken together, the CFA results therefore indicate a dominant general cognitive-performance component together with additional structure corresponding to the three theoretically specified content/performance dimensions. The correlated three-factor representation was retained for multidimensional IRT because it preserved the prespecified content organization without requiring the additional complexity of a bifactor parameterization. Table 9 compares the fit of the competing confirmatory factor and item-response models used to evaluate the dimensional structure of the assessment.

The IRT model comparisons favored the correlated three-dimensional specification over a unidimensional 2PL model. Allowing the three content/performance dimensions to correlate improved fit, Δ−2LL = 227.53, Δdf = 3, *p* < .001, and reduced both AIC and BIC. Estimated latent correlations ranged from 0.734 to 0.789, again indicating substantial shared performance variance. Adding bifactor-specific slopes improved −2LL by only 6.03 despite 21 additional parameters (*p* = .999) and increased AIC and BIC. The correlated multidimensional 2PL model was therefore retained as a useful content-domain representation, not as evidence that the three dimensions are independent cognitive abilities.

Item discrimination parameters in the selected model ranged from 0.889 to 2.497. Most scenarios showed moderate-to-high discrimination, indicating that they differentiated meaningfully among employees at different points on the relevant ability continuum. Item difficulty parameters ranged from −1.685 to 1.479, providing broad coverage of ability. S24 was the easiest item (*b* = −1.685), whereas S09 was the most difficult (*b* = 1.479). Approximate infit statistics ranged from 0.769 to .0946, with no item falling outside commonly acceptable ranges. Complete multidimensional IRT item parameters are reported in Table S7, with item characteristic and item information curves shown in Figures S1 and S2.

Table 10 presents the multidimensional IRT item parameters and related measurement-information statistics for the 24 retained scenarios.

Conditional-information analyses showed that measurement precision differed somewhat across the three dimensions. Cognitive control and adaptive reliance provided the greatest information, reaching a maximum of 5.22 around *θ* = −0.18, corresponding to a conditional standard error of 0.44. Evidence integration reached maximum information of 3.64 around *θ* = 0.03 (*SE* = 0.52), while AI error detection peaked at information = 3.42 around *θ* = 0.74 (*SE* = 0.54). Precision declined toward the tails of the ability distribution, particularly below *θ* = −2 and above *θ* = 2. Model-based marginal reliability was 0.787 for AI error detection, 0.801 for evidence integration, and 0.818 for cognitive control and adaptive reliance.

**Figure 3:**
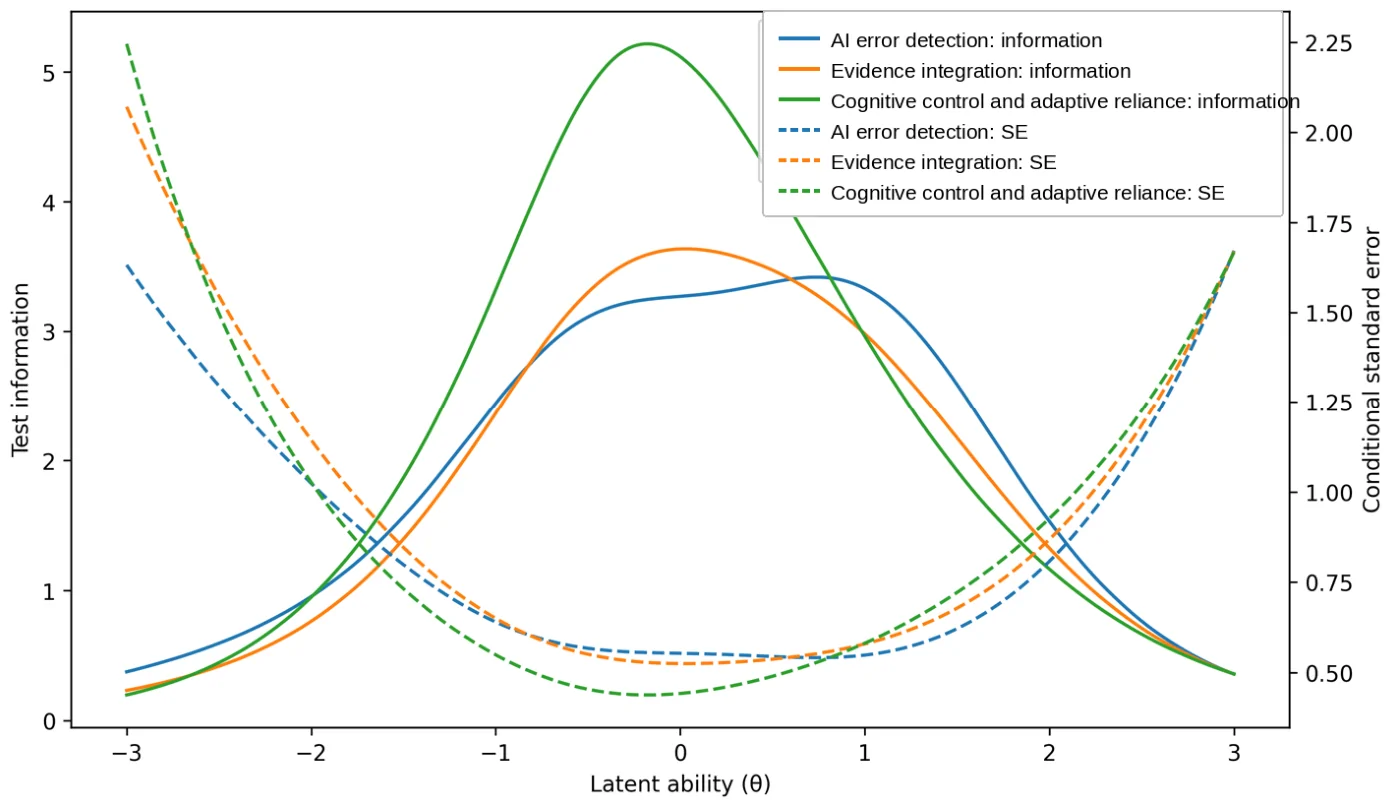
Test Information and Conditional Standard Error Across the Ability Continuum. Note. Dimension-specific curves are based on the correlated multidimensional 2PL representation and should not be interpreted as evidence of independent cognitive abilities.

Measurement precision was strongest through the low-to-moderately high portion of the performance continuum and declined toward the extremes. Broad item difficulties, generally strong discrimination, acceptable item fit, and model-based marginal reliabilities of 0.787–0.818 supported use of the correlated multidimensional 2PL model for item-level description while preserving the qualification that a strong general component underlies the three content/performance dimensions. Figure 3 presents the test information functions and corresponding conditional standard errors across the latent ability continuum for the three closely related content/performance dimensions. The figure illustrates where measurement precision is strongest and where it declines, providing a visual complement to the multidimensional IRT results reported above.

### 4.3. Accuracy, Response Time, Confidence, and Scenario-Level Associations

Accuracy and response time were examined jointly to determine whether higher performance reflected more efficient processing or merely greater time investment. At the person level, cognitive accuracy was positively associated with the item-adjusted processing-efficiency index, r = 0.249, *p* < .001. Thus, participants who performed more accurately also tended, on average, to respond somewhat more efficiently rather than requiring systematically longer processing time. At the item level, difficulty was strongly related to time intensity: more difficult scenarios produced longer response times, *B* = 0.646, *SE* = 0.095, 95% CI [0.448, 0.844], *p* < .001. Within participants and scenarios, correct responses were approximately 5.8% faster than incorrect responses, *B* = −0.060, *SE* = 0.004, 95% CI [−0.067, −0.053], *p* < .001.

The response-time pattern provided little evidence that rapid responding represented superficial engagement. Participants in the highest quartile of the processing-efficiency distribution achieved a mean accuracy of 0.544, compared with 0.452 among the remaining participants, *p* < .001. Faster responding therefore tended to accompany, rather than undermine, successful performance. These findings suggest that the assessment captured meaningful variation in cognitive efficiency: difficult items required more processing time, but higher-performing individuals were generally able to reach correct decisions with greater efficiency. Response-time screening and sensitivity analyses are reported in Table S8. Table 11 summarizes the joint accuracy-response-time estimates used to evaluate processing efficiency across participants and scenarios.

**Figure 4:**
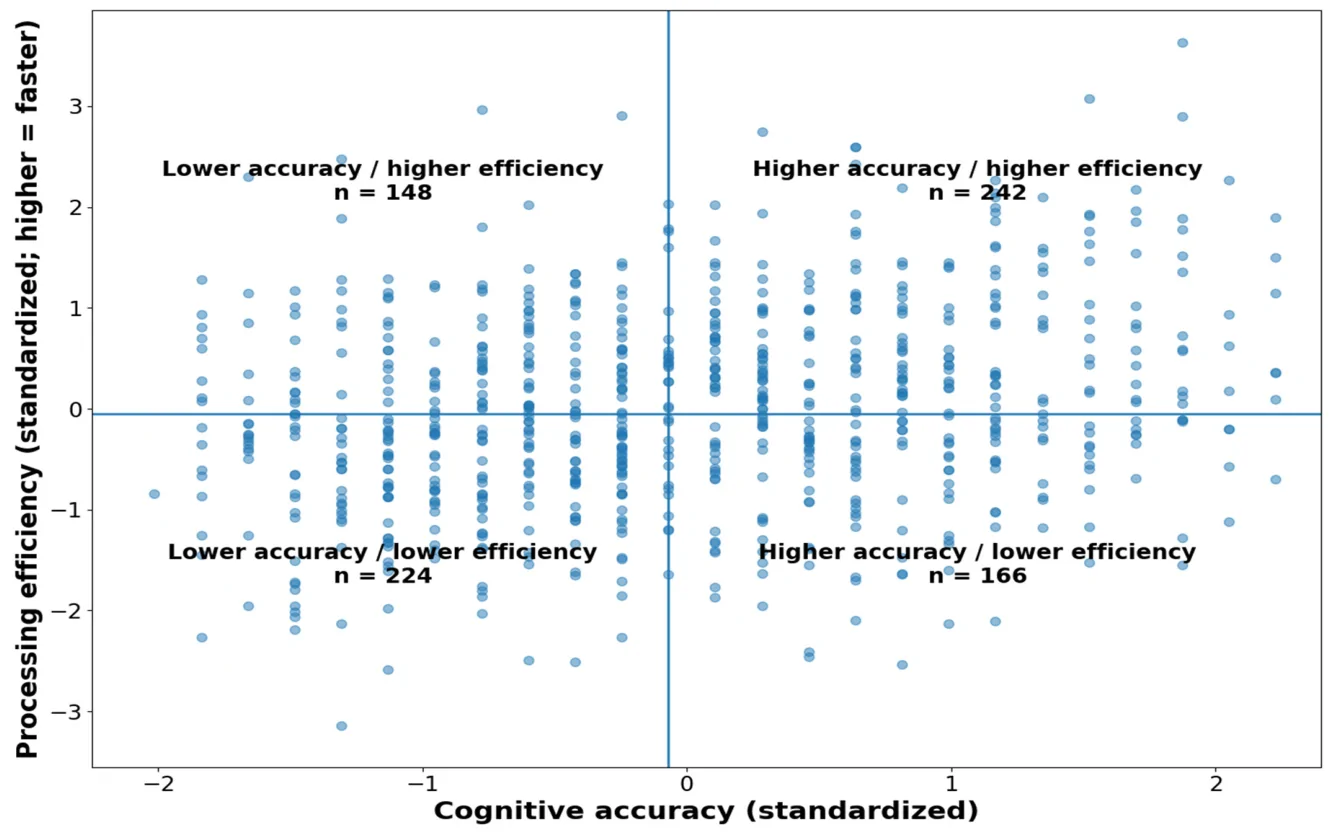
Relationship Between Cognitive Accuracy and Processing Efficiency.

Figure 4 illustrates the relationship between accuracy and processing efficiency. The median divisions are used only to make the distribution easier to visualize and do not represent empirically derived cognitive profiles. Of the 780 participants, 242 showed relatively high accuracy and high efficiency, 166 showed high accuracy with lower efficiency, 148 showed lower accuracy despite relatively higher processing efficiency, and 224 showed both lower accuracy and lower processing efficiency.

Final decision accuracy was examined using logistic regression with two-way participant- and scenario-clustered covariance estimation. Because only 24 scenario clusters were available, the primary inferential summary used a conservative t23 reference distribution. The primary model did not use the within-assessment 23-scenario rest score as a person-level ability predictor; instead, it included ICAR-16, working memory, processing speed, attentional control, and AI literacy together with the prespecified scenario characteristics and interactions. Scenario characteristics were fixed properties of the fractional design, so coefficients are interpreted as adjusted associations within the observed scenario set rather than isolated causal effects.

Correct AI advice was associated with substantially greater odds of a correct final decision, *B* = 1.844, OR = 6.32, 95% CI [3.22, 12.43], *p* < .001. High AI confidence was not independently associated with final accuracy, OR = 1.85, *p* = .213. The AI correctness × high-confidence interaction was negative but did not reach the 0.05 threshold under the conservative small-cluster inference, *B* = −1.171, OR = 0.31, 95% CI [0.09, 1.09], *p* = .066. Descriptively, accuracy under incorrect AI advice was lower for high-confidence than low-confidence advice (0.404 vs. 0.451), but this pattern is not treated as confirmatory support for H1. Accordingly, H1 was not supported.

Time pressure was positively associated with final accuracy as a main term, OR = 3.94, *p* = .009, but it also showed a strong negative interaction with AI correctness, *B* = −3.090, OR = 0.046, *p* < .001. Because time-pressure status was attached to particular scenarios, this crossover cannot be interpreted as an isolated effect of time pressure. More importantly, the hypothesis-specific analysis of inappropriate acceptance among incorrect-AI scenarios did not support H2: observed inappropriate acceptance was 35.1% without time pressure and 30.8% under time pressure, and the adjusted time-pressure association was essentially null (*B* ≈ −0.003, OR ≈ 1.00, *p* = .967). H2 was therefore not supported.

Interruption was not associated with final accuracy, OR = 1.05, *p* = .833, and the interruption × working-memory interaction was also nonsignificant, OR = 1.07, *p* = .288. In addition, only four scenarios contained an interruption and only one of those scenarios belonged to the evidence-integration grouping, limiting identification of the specific dimension-contingent claim in H3. High information conflict and high task complexity also showed nonsignificant adjusted main associations (*p* = .359 and *p* = .892, respectively). H3 was therefore not supported.

Among person-level predictors in the revised primary model, ICAR-16 was positively associated with final accuracy, *B* = 0.311, OR = 1.36, *p* < .001. Working memory was also positive, *B* = 0.100, OR = 1.10, *p* = .011, as were attentional control, *B* = 0.342, OR = 1.41, *p* < .001, and AI literacy, *B* = 0.155, OR = 1.17, *p* < .001. Processing speed was positive but did not reach the 0.05 threshold under t23 inference, OR = 1.06, *p* = .097. The original 23-scenario rest score remained strongly associated with focal-scenario accuracy in sensitivity analyses, but because it is derived from the same assessment it is interpreted as within-assessment cross-scenario consistency rather than independent cognitive validity.

Table 12 reports the revised primary two-way cluster-robust logistic model of scenario-level final decision accuracy.

Confidence calibration varied across AI-advice subsets. Overall mean confidence was 0.615 whereas observed accuracy was 0.475, producing a positive calibration bias of 0.141. Under correct AI advice, mean accuracy was 0.526 and mean confidence was 0.632 (Brier score = 0.198). Under incorrect low-confidence advice, accuracy was 0.451 and confidence was 0.559. The largest descriptive miscalibration occurred under incorrect high-confidence advice, where confidence remained 0.628 while accuracy was 0.404, yielding a calibration bias of 0.224 and a Brier score of 0.230.

Time-pressure scenarios showed mean accuracy of 0.460 and mean confidence of 0.569, corresponding to a calibration bias of 0.109. These calibration summaries are descriptive comparisons across fixed scenario subsets and should not be interpreted as causal effects of the scenario characteristics. Table 13 summarizes confidence-calibration indicators across the principal AI-advice scenario subsets.

**Figure 5:**
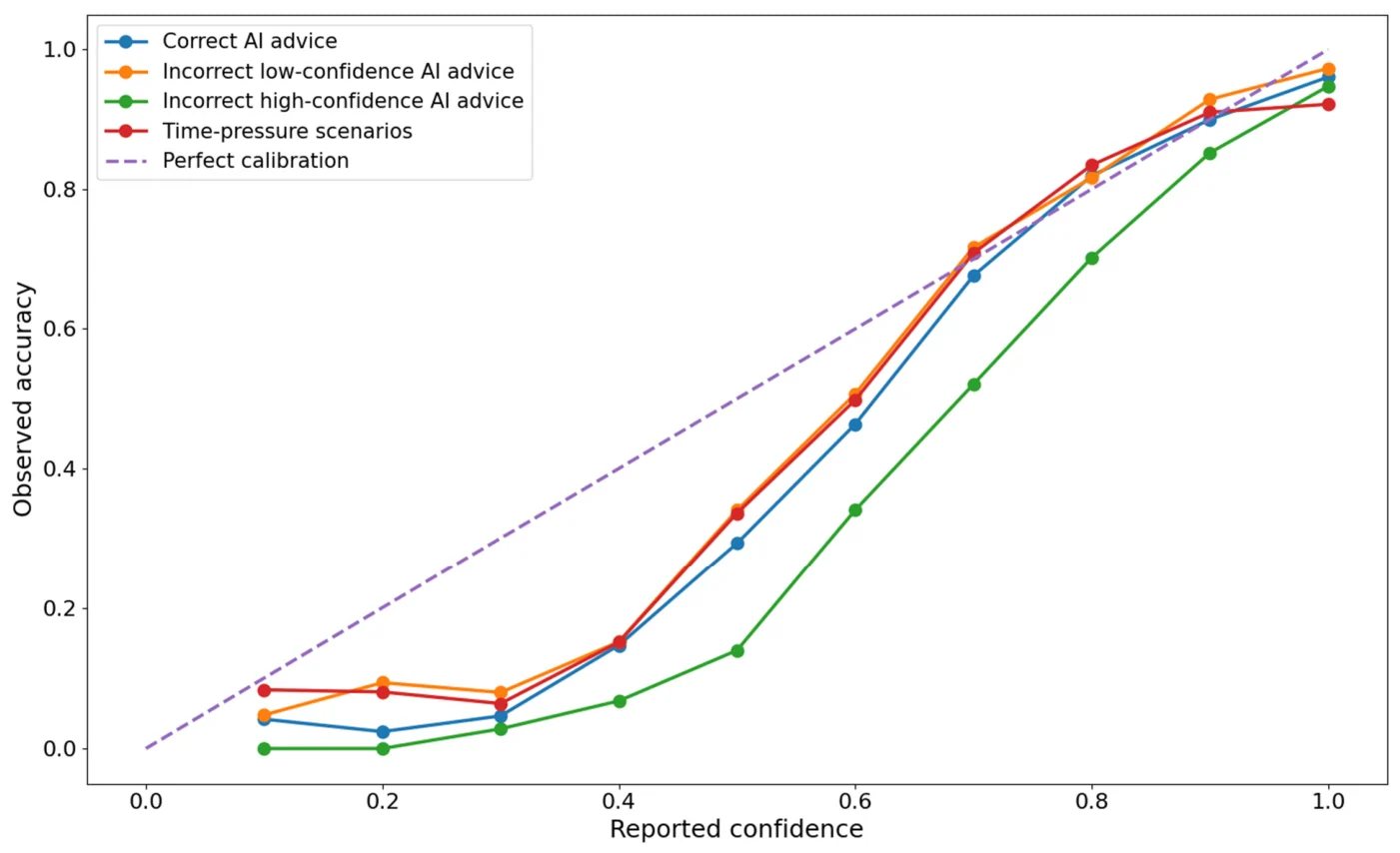
Confidence-Calibration Curves Across AI-Advice Scenario Subsets.

Figure 5 displays the confidence-calibration curves. Incorrect high-confidence advice showed the clearest descriptive tendency toward overconfidence. Because the corresponding correctness × confidence interaction in the revised primary accuracy model was not significant at α = 0.05 (*p* = .066), the calibration pattern is treated as descriptive evidence of misalignment between confidence and correctness rather than evidence that high AI confidence independently changed decision accuracy.

The scenario-level hypothesis tests therefore produced mixed evidence. H1 was not supported because the correctness × confidence interaction did not reach the 0.05 threshold. H2 was not supported because time pressure did not increase inappropriate acceptance of incorrect AI advice. H3 was not supported because interruption and the interruption × working-memory term were nonsignificant and the evidence-integration-specific claim was weakly identified by the fractional design. H5 was also not supported: among incorrect-AI scenarios, the high-confidence × overall-performance interaction was negative and nonsignificant, *B* = −0.269, OR = 0.764, *p* = .111, opposite to the predicted protective moderation. These results do not alter the separate psychometric and validity questions addressed below.

### 4.4. Construct, Concurrent Criterion-Related, and Incremental Validity

Convergent evidence was evaluated using both overall assessment performance and hypothesis-aligned content/performance scores. Final assessment accuracy correlated with ICAR-16 at r = 0.461, 95% CI [0.403, 0.514], working memory at r = 0.343, 95% CI [0.279, 0.403], processing speed at r = 0.259, 95% CI [0.193, 0.324], and attentional control at r = 0.440, 95% CI [0.382, 0.495], all *p* < .001. More directly aligned with H4, ICAR-16 correlated with the AI-error-detection score at r = 0.417, 95% CI [0.357, 0.473], *p* < .001, while working memory correlated with the evidence-integration score at r = 0.286, 95% CI [0.221, 0.350], *p* < .001. Cross-domain correlations were also positive, indicating that these mappings are theoretical emphases rather than exclusive one-to-one relationships. H4 was supported.

Relationships with AI-related self-reports were weaker. AI literacy was positively related to final performance, r = 0.246, 95% CI [0.179, 0.311], *p* < .001, and AI self-efficacy showed a smaller association, r = 0.161, 95% CI [0.092, 0.229], *p* < .001. Trust in AI was essentially unrelated to performance, r = −0.007, 95% CI [−0.077, 0.063], *p* = .841. Frequency of AI use was also weak and nonsignificant, Spearman’s ρ = 0.063, 95% bootstrap CI [−0.008, 0.134], *p* = .077. This pattern supports the distinction between demonstrated performance and AI-related self-perceptions or exposure.

Concurrent criterion-related validity was evaluated against the separately scored six-item organizational decision-quality task administered in the same session. Focal assessment performance correlated moderately with criterion performance, r = 0.408, 95% CI [0.348, 0.465], *p* < .001. Because the criterion was concurrent rather than prospective, this result is interpreted as concurrent criterion-related evidence and not as predictive validity. Table 14 summarizes convergent, discriminant, and concurrent criterion-related validity evidence.

Incremental concurrent validity was examined using a corrected hierarchical regression specification with organizational decision quality as the dependent variable. Model 1 contained demographic controls and explained *R*^2^ = 0.008 (adjusted *R*^2^ = −0.002). Adding work experience in Model 2 produced essentially no change, Δ*R*^2^ < 0.001, ΔF(1, 770) = 0.06, *p* = .808. Model 3 added AI-use frequency and prior generative-AI experience, increasing explained variance by 0.007, ΔF(2, 768) = 2.70, *p* = .068.

Model 4 added the conventional cognitive-performance block—ICAR-16, cognitive reflection, working memory, and processing speed—and increased explained variance by Δ*R*^2^ = 0.075, ΔF(4, 764) = 15.68, *p* < .001, yielding *R*^2^ = 0.090. Model 5 then added the AI-related self-report block comprising AI literacy, trust in AI, and AI self-efficacy. This block contributed little additional variance, Δ*R*^2^ = 0.003, ΔF(3, 761) = 0.92, *p* = .430.

Adding the focal 24-scenario assessment in Model 6 produced the largest incremental gain, Δ*R*^2^ = 0.103, ΔF(1, 760) = 97.04, *p* < .001. The final model explained *R*^2^ = 0.196 of concurrent organizational decision-quality variance (adjusted *R*^2^ = 0.176). Each additional correctly solved focal scenario was associated with a 0.129-point increase in criterion performance, *B* = 0.129, *SE* = 0.013, 95% CI [0.103, 0.155], *p* < .001; the standardized coefficient was β = 0.379. A sensitivity model that entered attentional control separately before the self-report block still showed a substantial focal-assessment increment, Δ*R*^2^ = 0.087, *p* < .001. Table 15 summarizes the corrected hierarchical regression models for incremental concurrent validity.

Internal 10-fold cross-validation showed the same general pattern. Models containing only demographic and experience variables had essentially no held-out explanatory value. The conventional cognitive block increased cross-validated *R*^2^ to 0.059, the AI-related self-report block did not improve it (CV *R*^2^ = 0.055), and adding the focal assessment increased CV *R*^2^ to 0.160 while reducing RMSE to 1.764. These values support internal statistical stability but do not constitute prospective prediction in a new population.

Overall, the validity analyses were stronger than the scenario-specific hypothesis tests. H4 was supported by dimension-aligned cognitive associations, and H6 was supported by a focal-assessment increment of Δ*R*^2^ = 0.103 in the concurrent criterion model. In contrast, H1, H2, H3, and H5 were not supported under the revised hypothesis-aligned tests. The final hypothesis pattern was therefore H1: not supported; H2: not supported; H3: not supported; H4: supported; H5: not supported; H6: supported.

### 4.5. Measurement Invariance and Differential Item Functioning

Measurement invariance was examined across gender, age, education, managerial status, AI-use frequency, and occupational grouping using the correlated three-factor representation as the reference measurement model. For binary responses, configural and loading invariance were evaluated before threshold invariance. Residual invariance was not separately estimated because residual variances are not freely identified in the same manner under the categorical specification. These analyses are interpreted as preliminary group-comparability evidence, not as proof of fairness for consequential assessment use.

Configural and loading invariance results were broadly favorable across the six comparisons. Configural CFI values ranged from 0.975 to 0.992 and RMSEA values from 0.009 to 0.015. Equality constraints on factor loadings produced small changes in fit, with absolute ΔCFI values from 0.000 to 0.005 and ΔRMSEA values from 0.000 to 0.002.

Threshold invariance was supported for gender, age, education, managerial status, and AI-use frequency. The occupational comparison was the exception: constraining thresholds reduced CFI from 0.974 to 0.960 (ΔCFI = −0.014) and increased RMSEA from 0.015 to 0.019 (ΔRMSEA = 0.003), and the joint threshold-difference test was significant, χ^2^(24) = 75.66, *p* < .001. Full occupational threshold invariance was therefore not supported. Cross-occupation score comparisons should consequently be interpreted cautiously until this finding is replicated.

Table 16 presents the measurement-invariance results across the examined groups.

Differential item functioning was evaluated by logistic regression using performance on the remaining 23 items as the matching score. Uniform DIF was represented by the group term and non-uniform DIF by the group × matching-score interaction. Benjamini–Hochberg false-discovery-rate adjustment was applied separately to the uniform- and non-uniform-DIF families within each comparison. Practical importance was evaluated using ΔMcFadden *R*^2^ and the adjusted probability difference; values below approximately 0.02 were treated as small.

No item survived FDR correction for gender, age, education, managerial status, or occupational group. The largest observed item-level effects in these comparisons were small. Importantly, the absence of an individual occupational item surviving FDR correction does not negate the aggregate threshold-invariance result reported above; the two procedures address different aspects of group comparability.

One item, S01, showed significant uniform DIF across AI-use-frequency groups after FDR correction (q = 0.021, ΔMcFadden *R*^2^ = 0.011). At comparable matching-score levels, participants using AI weekly or less had an adjusted probability of correct performance approximately 0.101 lower than participants using AI daily or more frequently. The magnitude was small, so S01 was retained but should be monitored in future samples.

Table 17 summarizes the differential item functioning results across the examined groups.

Planning/analytical employees had a higher mean total score than operations-oriented employees (11.96 vs. 10.79, *p* = .004). This mean difference does not explain away the unfavorable occupational threshold invariance result. Although no individual occupational item survived FDR correction, the aggregate threshold test remained unfavorable; raw cross-occupation comparisons should therefore remain provisional. At the item level, the detected DIF effects were generally small and no item required deletion or group-specific scoring in this sample. However, item-level DIF and measurement-invariance results should be considered jointly. The occupational threshold finding prevents a broad claim of equivalent functioning across all examined groups. Overall, the analyses provide preliminary evidence of broadly similar measurement functioning across gender, age, education, managerial status, and AI-use-frequency groups, with an important occupational qualification. Full threshold invariance was not supported across occupational groups, and S01 showed a small AI-use-related DIF effect. These findings support continued psychometric evaluation and replication rather than claims that group comparability or fairness has been definitively established.

## 5. Discussion

### 5.1. Interpretation of the Main Findings

The purpose of this study was to examine whether cognitive performance under AI advice can be assessed as a meaningful applied performance domain in organizational decision-making. The results provide stronger support for the study’s psychometric and validity objectives than for several of its scenario-specific hypotheses. H4 and H6 were supported, whereas H1, H2, H3, and H5 were not supported under the revised hypothesis-aligned tests. This pattern does not imply that the assessment was unsuccessful. Reliability, item functioning, dimensionality, cognitive convergence, concurrent criterion-related validity, and incremental validity address different questions from the scenario-level hypotheses. The findings therefore support an initial measurement framework while indicating that several proposed contextual mechanisms require stronger experimental designs before they can be treated as established features of performance under AI advice.

The dimensionality results support a qualified interpretation. The one-factor CFA fit poorly, whereas the correlated three-factor model represented the data substantially better; however, its CFI (0.896) and TLI (0.885) remained below commonly preferred levels, and the three factors correlated strongly. Higher-order and bifactor analyses likewise indicated a dominant general cognitive-performance component. The most defensible interpretation is therefore not that AI error detection, evidence integration, and cognitive control and adaptive reliance are independent abilities, but that they are closely related content/performance dimensions embedded within a strong general performance structure. The multidimensional IRT results add useful content-level information, yet they should not be treated as proof of sharply distinct latent traits.

The convergent-validity pattern is consistent with the CHC-informed rationale. Overall performance was positively associated with ICAR-16, working memory, processing speed, and attentional control, while the dimension-aligned tests showed that ICAR-16 was associated with AI error detection (r = 0.417) and working memory with evidence integration (r = 0.286), both *p* < .001. These results support H4, while the positive cross-domain correlations also show that the theoretical mappings are not exclusive. Fluid reasoning and working memory appear to contribute across multiple task demands rather than map one-to-one onto a single content grouping. Attentional control should likewise be interpreted as a companion individual-difference resource rather than as evidence for a newly established CHC dimension of the assessment.

The process indicators provided complementary information. Higher-performing participants tended to respond somewhat more efficiently, and more difficult scenarios required more time, suggesting that response speed is informative only when interpreted jointly with accuracy. Confidence calibration showed a different pattern: confidence exceeded observed accuracy overall, and the largest descriptive miscalibration occurred when incorrect AI advice was expressed with high confidence. This pattern is consistent with concerns about reliance calibration in human–AI decision-making (Dogru & Krämer, 2025; Lee & See, 2004; Meincke et al., 2026; Pearson et al., 2026), but it should not be overstated. The correctness × confidence interaction did not reach the 0.05 threshold under conservative small-cluster inference (*p* = .066), so H1 was not supported and the calibration pattern is best treated as descriptive evidence of confidence–accuracy misalignment.

The remaining scenario-specific hypotheses also require restraint. Time pressure did not increase inappropriate acceptance of incorrect AI advice as predicted in H2, and interruption was not significantly associated with accuracy or with the interruption × working-memory interaction, so H3 was not supported. H5 was also not supported: higher overall performance did not significantly reduce susceptibility to confidently expressed inaccurate AI recommendations in the hypothesis-specific moderation analysis. The strong correctness × time-pressure crossover in the accuracy model is therefore interpreted as an association within the observed scenario set rather than confirmation of H2. Because confidence, time pressure, interruptions, information conflict, and complexity were fixed characteristics of particular scenarios in the fractional design rather than independently randomized within otherwise identical content, these coefficients cannot isolate causal effects from scenario content.

The clearest evidence for the assessment’s added value comes from the validity analyses. Performance correlated moderately with the concurrently administered organizational decision-quality criterion (r = 0.408) and explained an additional 10.3% of criterion variance after demographic and work characteristics, AI experience, conventional cognitive-performance measures, and AI-related self-reports were entered (Δ*R*^2^ = 0.103, *p* < .001). H6 was therefore supported. The self-report block itself added little unique variance (Δ*R*^2^ = 0.003, *p* = .430). This pattern suggests that the focal assessment captures applied performance information not reducible to conventional cognition or AI-related self-perceptions. At the same time, the criterion was measured in the same session, so the evidence is concurrent rather than predictive. Likewise, the 23-scenario rest score is best viewed as a within-assessment cross-scenario consistency indicator and is retained only as a sensitivity analysis, not as independent validity evidence.

### 5.2. Theoretical and Measurement Contributions

The principal contribution is psychometric and integrative. Behavioral research on automation, advice taking, and human–AI reliance already examines whether people accept, reject, or revise machine advice; the present study does not claim to introduce behavioral measurement itself. Instead, it organizes repeated workplace-relevant AI-advice decisions into an individual-differences assessment framework that connects scenario performance with cognitive measures, process indicators, criterion-related evidence, item-response modelling, and group-comparability analyses. This framing sharpens the contribution relative to prior reliance paradigms by treating repeated behavioral performance as an assessment problem rather than only as a situational outcome.

The CHC contribution should also be understood as application rather than theoretical extension. CHC theory offers a well-established vocabulary for reasoning, working memory, and processing efficiency, allowing the cognitive demands of AI-supported decisions to be described without proposing a new broad intelligence. The results are compatible with that position: performance shared meaningful variance with established cognitive measures, yet the focal assessment also captured additional applied variance in a decision environment that requires evaluating machine-generated advice. Accordingly, cognitive performance under AI advice is best described as a CHC-informed applied performance domain rather than a new CHC ability or a distinct form of “AI intelligence.”

A second contribution concerns the separation of demonstrated performance from AI-related self-perceptions. AI literacy and AI self-efficacy were positively but more weakly associated with performance, trust in AI was essentially unrelated to final accuracy, and AI-use frequency showed only a weak nonsignificant relationship. These constructs remain important for understanding adoption, confidence, and engagement, but they do not substitute for observing whether a person detects an error, integrates evidence, or relies appropriately on a recommendation. The assessment therefore complements, rather than replaces, established AI-literacy, trust, and self-efficacy measures (Carolus et al., 2023; Jian et al., 2000; Koch et al., 2024; Wang & Chuang, 2024).

A third contribution is the combination of outcome and process information. Final accuracy provides the core performance score, while AI-evaluation accuracy, reliance behavior, confidence, decision revision, and response time help characterize how decisions were reached. Multidimensional IRT describes where scenarios provide information across the performance continuum, and internal cross-validation assesses statistical stability of the incremental models. The two-way cluster-robust scenario analysis additionally recognizes dependence within participants and scenarios, with conservative t23 inference used because only 24 scenario clusters were available. These methods provide complementary evidence, although none by itself establishes construct validity or causal mechanisms.

The group-comparability analyses provide an important qualification to the measurement contribution. Loading and threshold invariance were broadly favorable for gender, age, education, managerial status, and AI-use frequency, and item-level DIF effects were generally small. However, full occupational threshold invariance was not supported (ΔCFI = −0.014), even though no single occupational item survived FDR correction. The aggregate threshold result should not be dismissed because of the item-level DIF pattern. Cross-occupation score comparisons therefore require caution until the finding is replicated, and the present analyses should be described as preliminary group-comparability evidence rather than proof of fairness.

More broadly, the study provides initial validation evidence from one sample and one administration. The psychometric results are sufficiently coherent to justify continued development and independent replication, but they do not establish a finalized high-stakes instrument. The combination of a dominant general component, useful content-specific structure, convergent cognitive relationships, concurrent criterion-related evidence, and incremental validity is promising precisely because the conclusions can be stated without requiring every scenario-specific hypothesis to be confirmed.

### 5.3. Organizational and Practical Implications

The most defensible near-term applications are research, formative assessment, employee development, and training evaluation rather than personnel selection. Organizations often evaluate AI training through completion, satisfaction, or self-reported confidence. A performance-based assessment could complement those indicators by examining whether employees become better at evaluating inaccurate recommendations, integrating task evidence, regulating reliance, and calibrating confidence. Because the present study provides initial rather than definitive validation, such uses should focus on learning and development rather than consequential classification of employees.

The results also suggest that AI-literacy development may benefit from separating knowledge from performance. Employees can understand general AI limitations yet still struggle with verification in a concrete decision, while frequent AI use does not necessarily imply better judgment. Future training studies could use the assessment to test whether targeted verification routines, evidence-integration exercises, or calibration feedback improve performance. Such intervention effects were not tested here, so these possibilities should be treated as directions for applied research rather than established prescriptions.

For decision-support design, the calibration results highlight the importance of how confidence and uncertainty are communicated. Incorrect high-confidence advice was associated descriptively with the greatest confidence–accuracy mismatch, but the corresponding interaction with final accuracy was not statistically significant at α = 0.05. The practical implication is therefore not that confidence displays have been shown to cause overreliance, but that interface designs should be evaluated for whether they support verification and calibrated reliance. Explanations that expose evidence, assumptions, uncertainty, and omitted information may be more useful than confidence cues presented without context.

At the workflow level, organizations should evaluate the interaction between system characteristics, human capabilities, and decision context rather than focus only on AI accuracy (Do Khac & Leyer, 2026; Gonzalez & Heidari, 2025; Li & Tian, 2026). The current fractional design does not establish that time pressure or interruptions causally reduce or improve performance, so workflow recommendations about these factors should await stronger within-scenario experiments. Nevertheless, the framework offers a way to study where oversight becomes cognitively demanding and where employees may need clearer evidence, more verification support, or better-calibrated system communication.

The assessment should not currently be used as a stand-alone basis for hiring, promotion, compensation, discipline, termination, or other high-stakes personnel decisions. Such applications would require substantially stronger evidence, including independent replication, prospective criterion validity, test–retest reliability, alternate forms, accessibility evaluation, adverse-impact analysis, cross-cultural and occupational comparability, and validation across different AI systems. The occupational threshold non-invariance observed here is an additional reason to avoid consequential cross-group interpretations at this stage.

### 5.4. Limitations and Future Research

Several limitations define the boundaries of the findings. First, the 24-scenario design was fractional: scenario characteristics were fixed to particular scenarios rather than independently crossed within otherwise identical content. Randomization applied to presentation order, not to independent assignment of confidence, time pressure, interruption, information conflict, or complexity. Scenario-level coefficients therefore represent adjusted associations within this scenario set and cannot establish causal effects. The small scenario-cluster dimension (24 scenarios) further limits precision, despite the conservative t23 inference and leave-one-scenario-out sensitivity analyses. Future work should use larger scenario banks and fully or partially crossed experimental manipulations when the goal is to identify contextual mechanisms.

Second, the study provides initial validation evidence from a single Turkish sample recruited through purposive nonprobability procedures and assessed once. This design limits claims about population generalizability, temporal stability, and cross-cultural equivalence. Test–retest studies, alternate forms, independent calibration samples, probability-based or more broadly recruited samples, and replications in other languages and countries are needed before the assessment can be treated as a stable individual-differences instrument.

Third, online administration introduces variability in device, screen size, connectivity, distraction, and testing environment. Response-time interpretation is especially sensitive to these factors. Timing information was derived from the study’s supplementary timestamping procedure rather than from a standardized laboratory platform, and very slow responses were not automatically discarded because they could reflect genuine processing difficulty. Future studies should archive detailed timing metadata and compare supervised with unsupervised administration to separate cognitive processing time from technical delay more precisely.

Fourth, the companion cognitive measures were intentionally brief to limit participant burden. The retained dataset contains aggregate working-memory and processing-speed scores rather than complete trial-level records, and attentional control was represented by eight study-specific binary indicators rather than a standardized CHC test. These measures were useful as correlates and sensitivity predictors, but future replications should employ fully documented standardized tasks, preserve trial-level data, and evaluate their reliability and validity independently. This is particularly important for reproducing the exact cognitive-measurement component of the study.

Fifth, the criterion evidence is concurrent and limited in scope. The six-item organizational decision-quality task was administered in the same session as the focal assessment, so the observed r = 0.408 and incremental Δ*R*^2^ = 0.103 do not demonstrate prediction of future workplace performance. Future studies should examine supervisor ratings, objective errors, forecasting performance, quality-control outcomes, and real AI-assisted decisions collected prospectively. Similarly, internal 10-fold cross-validation evaluates statistical stability within the present dataset and is not a substitute for external validation in a new sample.

Sixth, group comparability requires further study. Full occupational threshold invariance was not supported, and S01 showed a small AI-use-related DIF effect. Although the detected item-level effects were generally small, these results are sufficient to caution against strong claims of fairness or interchangeable score meaning across occupations. Larger subgroup samples, replication across industries, accessibility studies, and analyses of practical adverse impact are needed before cross-group comparisons can support consequential decisions.

Seventh, the within-assessment rest score used in earlier scenario-level analyses is not an independent measure of cognitive ability because it is derived from performance on the same 24-scenario assessment. Excluding the focal item prevents direct arithmetic overlap but does not create an external validity criterion. The revised primary model therefore relies on independently measured person-level predictors, while the rest score is retained only as an internal cross-scenario consistency sensitivity analysis. Future work should prioritize independent cognitive and behavioral predictors when testing person-level moderation.

Finally, both AI systems and workplace uses of AI are evolving rapidly. Scenario realism, expectations of system reliability, and the meaning of appropriate reliance may change as interfaces and models develop. Periodic content review will therefore be necessary. Future research should also test different AI systems, industry-specific scenarios, longitudinal training responsiveness, and real workplace deployment, while examining whether the same general performance structure emerges across contexts.

Further psychometric development could expand the item bank and evaluate computerized adaptive testing, particularly because item information varied across the performance continuum. Such work should follow, rather than precede, independent replication of the factor structure, stronger documentation of companion cognitive tasks, prospective criterion validation, and additional group-comparability evidence.

Overall, the study supports a restrained interpretation. Cognitive performance under AI advice is best understood as a CHC-informed applied performance domain with a dominant general component and meaningful content-specific structure. The central psychometric and validity objectives received stronger support than the proposed scenario-specific mechanisms. That distinction is informative: the assessment shows promise as a research and developmental instrument for observing how people evaluate and respond to AI advice, while the contextual moderators, temporal stability, external validity, and cross-group comparability require further independent study.

## 6. Conclusions

This study developed and initially validated a CHC-informed, performance-based assessment of cognitive performance under AI advice using repeated organizational decision scenarios. The findings do not support interpreting performance under AI advice as a new form of intelligence. Instead, they indicate a dominant general cognitive-performance component together with additional structure corresponding to three closely related content/performance dimensions: AI error detection, evidence integration, and cognitive control and adaptive reliance. This pattern is consistent with the view that established cognitive resources are expressed within a distinctive decision environment in which employees must evaluate the quality and usefulness of machine-generated advice.

The strongest evidence concerned the assessment’s psychometric and validity objectives. Performance showed meaningful relationships with established cognitive measures, and the dimension-aligned analyses supported H4: ICAR-16 was associated with AI error detection and working memory with evidence integration. Focal assessment performance was also moderately related to the concurrently administered organizational decision-quality task (r = 0.408) and explained additional concurrent criterion variance beyond demographic and work characteristics, AI experience, conventional cognitive-performance measures, and AI-related self-reports (Δ*R*^2^ = 0.103), supporting H6. These findings suggest that the assessment captures applied performance that is related to, but not reducible to, conventional cognitive measures or self-reported AI competence.

The scenario-specific hypotheses produced more limited evidence. H1, H2, H3, and H5 were not supported under the revised hypothesis-aligned analyses. In particular, the confidence interaction did not reach the conventional significance threshold, time pressure did not increase inappropriate acceptance of incorrect AI advice, the interruption hypothesis was not supported, and higher overall assessment performance did not provide the predicted protection against confidently expressed inaccurate advice. Because scenario characteristics were fixed properties of a 24-scenario fractional design rather than independently crossed manipulations, these results should be treated as adjusted associations within the observed scenario set. Stronger fully or partially crossed designs are needed before these contextual mechanisms can be established.

Accordingly, the contribution of the study is primarily psychometric and integrative rather than the introduction of behavioral AI-reliance measurement itself. The assessment combines final accuracy with AI-evaluation accuracy, reliance behavior, confidence, response time, and decision revision, while item-response and process analyses provide information about measurement precision and how performance unfolds. The present evidence should nevertheless be considered initial. It comes from one purposively recruited Turkish sample and one administration; the criterion evidence is concurrent rather than prospective; the scenario design contains only 24 fixed scenario clusters; and full occupational threshold invariance was not supported. The assessment is therefore best suited at present to research, formative assessment, and developmental or training contexts rather than hiring, promotion, or other consequential personnel decisions. Future work should establish test–retest reliability, prospective criterion validity, independent replication, cross-cultural comparability, stronger documentation of companion cognitive tasks, accessibility, and group comparability before higher-stakes applications are considered.

## Figures and Tables

**Table 1:** Cognitive Foundations and Proposed Assessment Framework.

Resource	Theoretical Function	Framework Role	Task Behavior	Indicator
Fluid reasoning (CHC)	Evaluates relationships, draws inferences, and solves unfamiliar problems	AI error detection	Identifies errors, unsupported claims, contradictions, or misleading conclusions	Error-detection accuracy; final accuracy
Working memory (CHC)	Maintains and integrates multiple pieces of information	Evidence integration	Combines AI advice with numerical information, policies, context, and constraints	Evidence-integration accuracy; decision revision
Processing speed (CHC)	Processes relevant information efficiently	Processing efficiency	Evaluates information efficiently without sacrificing accuracy	Response time with accuracy
Attentional control *	Maintains task goals and resists distracting or misleading cues	Cognitive control and adaptive reliance	Resists automatic acceptance and re-evaluates advice when needed	Appropriate reliance; accuracy under interruption
Performance monitoring	Monitors the quality and certainty of one’s own judgment	Confidence calibration	Adjusts certainty to the strength and correctness of the decision	Confidence–accuracy correspondence; calibration error

Note. * Attentional control is treated as a companion individual-difference resource rather than as a separately established CHC dimension of the assessment. Taken together, the framework treats cognitive performance under AI advice as an applied performance domain with a potentially strong general component and additional content-specific structure. It does not propose a new form of intelligence or assume that the three dimensions are psychometrically independent. The validity question is instead whether scenario-based performance shows coherent measurement properties, meaningful relationships with established cognitive measures, weaker or distinct relationships with AI-related self-reports, and incremental concurrent association with organizational decision quality.

**Table 2:** Assessment Blueprint and Organizational Scenario Structure.

Content/Performance Dimension	Organizational Task Context	Cognitive Requirement	Scenario Characteristic	Required Response	Process Indicator
AI error detection	Risk, quality, supplier, customer decisions	Detect inaccurate or unsupported advice	Correct/incorrect; high/low confidence	Evaluate AI advice and decide	Error-detection and final accuracy
Evidence integration	Inventory, budget, scheduling, planning	Combine evidence and constraints	Consistent/conflicting advice	Integrate information and decide	Accuracy and decision revision
Cognitive control and adaptive reliance	Logistics, project, operational decisions	Resist automatic acceptance and reconsider judgments	Correct/incorrect advice; pressure/interruption	Accept, reject, or revise	Appropriate reliance and revision
Cross-cutting confidence calibration	All contexts	Align certainty with correctness	High/low AI confidence	Rate confidence	Confidence–accuracy calibration
Cross-cutting processing efficiency	All contexts	Evaluate evidence efficiently	All conditions	Complete decision task	Response time with accuracy

**Table 3:** Scenario Characteristics Embedded in the Organizational Decision Tasks.

Scenario Characteristic	Levels	Allocation	Expected Cognitive Demand/Association	Primary Outcome
AI-advice correctness	Correct/incorrect	12/12	Requires discrimination of valid and faulty advice	Final accuracy; appropriate reliance
AI confidence	High/low	12/12	May shape acceptance independently of correctness	Inappropriate acceptance; calibration
Time pressure	Present/absent	6/18	Limits time available for verification	Accuracy; response time; reliance
Interruption	Present/absent	4/20	Disrupts attention and information maintenance	Accuracy; revision behavior
Information conflict	High/low	12/12	Increases evidence-integration demand	Evidence-integration performance
Task complexity	Low/moderate/high	8/8/8	Increases coordination and reasoning demands	Final accuracy

**Table 4:** Content Validation and Pilot Item Selection.

Item/Group	I-CVI	CVR	Pilot Difficulty	Pilot Discrimination	Mean Response Time	Final Decision
23 retained items	0.80–1.00	0.60–1.00	0.275–0.783	0.290–0.676	42.4–47.4 s	Retain
I06	0.80	0.60	0.717	0.204	45.3 s	Revise and retain
I25–I30	0.00–0.60	−1.00–0.20	0.267–0.467	0.098–0.330	55.6–63.2 s	Remove

**Table 5:** Main-Sample Demographic, Occupational, and AI-Use Characteristics.

Characteristic	Category/Statistic	*n*	%/*M* (*SD*)
Final sample	—	780	100.0
Age	Range = 20–64	780	38.82 (9.31)
Work experience	Range = 1–34 years	780	10.50 (5.29)
Gender	Male	427	54.7
	Female	348	44.6
	Nonbinary/Other	5	0.6
Education	High school	62	7.9
	Associate	87	11.2
	Bachelor	349	44.7
	Master	279	35.8
	Doctoral	3	0.4
Managerial status	Manager	234	30.0
	Nonmanager	546	70.0
AI-use frequency	Several times daily	118	15.1
	Daily	315	40.4
	Weekly	202	25.9
	Monthly	91	11.7
	Rarely	54	6.9

**Table 6:** Measures, Scoring, Reliability, and Analytic Roles.

Measure/Construct	Items/Tasks	Response/Scoring	Reliability	Analytic Role	Source/Status
Cognitive performance under AI advice	24 scenarios	Final accuracy 0–24; AI-evaluation accuracy; reliance; calibration; RT; revision	α = 0.877 for final accuracy	Primary focal assessment	Study-developed
ICAR-16 reasoning	16 items	Correct/incorrect; sum 0–16	α = 0.809	Convergent validity; primary scenario predictor	(Condon & Revelle, 2014; Dworak et al., 2021)
Cognitive reflection	4 items	Correct/incorrect; sum 0–4	α = 0.542	Convergent validity; incremental block	(Frederick, 2005; Thomson & Oppenheimer, 2016)
Working memory	Brief performance task	Accuracy-based total; observed 3–14; higher = stronger performance	Not estimable from retained aggregate data	Convergent validity; scenario predictor	(Conway et al., 2005; Unsworth et al., 2005)
Processing speed	Brief timed task	Performance total; observed 30–80; higher = stronger performance	Not estimable from retained aggregate data	Convergent validity; scenario predictor	Study companion performance task
Attentional control	8 indicators	Binary 0/1; sum 0–8	α = 0.718	Companion predictor; sensitivity analysis	Study-specific ATT1–ATT8
AI literacy	MAILS-10	Six-point items; mean score	α = 0.914	Related self-report; incremental block	(Carolus et al., 2023; Koch et al., 2024)
Trust in AI	12 items	Six-point items; mean score	α = 0.921	Related self-report; incremental block	(Jian et al., 2000)
AI self-efficacy	6 items	1–6 items; mean score	α = 0.873	Related self-report; incremental block	Study measure; Supplementary S6
AI-use frequency	1 item	Rarely (1) to several times daily (5)	—	Experience control; group comparison	Study demographic item
Prior generative-AI experience	1 item	Limited (1), Moderate (2), Extensive (3)	—	Experience control	Study demographic item
Organizational decision quality	6 items	Correct/incorrect; sum 0–6	α = 0.748	Concurrent criterion-related validity	Study criterion task

**Table 7:** Descriptive Statistics and Correlations Among the Principal Study Variables.

No.	Variable	*M*	*SD*	α	1	2	3	4	5	6	7	8	9	10	11	12
1	Final accuracy	11.40	5.66	0.877	—											
2	AI-evaluation accuracy	11.10	3.25	—	0.59 ***	—										
3	Appropriate reliance	14.98	3.01	—	0.65 ***	0.33 ***	—									
4	ICAR-16	7.80	3.86	0.809	0.46 ***	0.31 ***	0.25 ***	—								
5	Cognitive reflection	1.76	1.25	0.542	0.34 ***	0.23 ***	0.18 ***	0.50 ***	—							
6	Working memory	7.96	2.12	—	0.34 ***	0.22 ***	0.22 ***	0.44 ***	0.29 ***	—						
7	Processing speed	55.14	9.34	—	0.26 ***	0.18 ***	0.20 ***	0.32 ***	0.24 ***	0.29 ***	—					
8	Attentional control	3.94	2.24	0.718	0.44 ***	0.37 ***	0.25 ***	0.28 ***	0.25 ***	0.26 ***	0.20 ***	—				
9	AI literacy	3.50	1.05	0.914	0.25 ***	0.19 ***	0.15 ***	0.20 ***	0.13 ***	0.16 ***	0.17 ***	0.08 *	—			
10	Trust in AI	3.53	1.01	0.921	−0.01	−0.01	0.03	0.05	−0.01	0.04	0.01	0.04	0.06	—		
11	AI self-efficacy	3.55	1.35	0.873	0.16 ***	0.09 **	0.11 **	0.08 *	0.04	0.12 **	0.11 **	0.05	0.59 ***	0.13 ***	—	
12	Criterion performance	2.99	1.93	0.748	0.41 ***	0.29 ***	0.24 ***	0.26 ***	0.18 ***	0.19 ***	0.14 ***	0.21 ***	0.06	0.02	0.00	—

Note. *N* = 780. AI-evaluation accuracy and appropriate reliance are process indicators derived from the scenario tasks; internal-consistency coefficients are therefore not reported for these variables. * *p* < 0.05; ** *p* < 0.01; *** *p* < 0.001.

**Table 8:** Preliminary Item Performance and Retention Summary.

Item	Assessment Dimension	Difficulty	Discrimination	Corrected Item–Total r	Mean RT (s)	Status
S01	Cognitive control and adaptive reliance	0.449	0.754	0.533	38.75	Retain
S02	Cognitive control and adaptive reliance	0.327	0.616	0.480	34.46	Retain
S03	Evidence integration	0.533	0.711	0.491	39.34	Retain
S04	Cognitive control and adaptive reliance	0.622	0.687	0.495	33.16	Retain
S05	AI error detection	0.241	0.569	0.469	46.70	Retain
S06	AI error detection	0.263	0.545	0.435	38.81	Retain
S07	AI error detection	0.406	0.592	0.427	34.26	Retain
S08	Evidence integration	0.685	0.445	0.324	34.69	Retain
S09	Cognitive control and adaptive reliance	0.181	0.379	0.367	43.93	Retain
S10	Evidence integration	0.412	0.564	0.400	34.98	Retain
S11	AI error detection	0.596	0.706	0.493	31.97	Retain
S12	Evidence integration	0.487	0.640	0.446	37.76	Retain
S13	Evidence integration	0.273	0.583	0.474	38.54	Retain
S14	AI error detection	0.682	0.654	0.498	26.86	Retain
S15	AI error detection	0.682	0.412	0.312	33.07	Retain
S16	AI error detection	0.201	0.512	0.473	42.75	Retain
S17	Cognitive control and adaptive reliance	0.791	0.507	0.413	31.65	Retain
S18	Evidence integration	0.459	0.659	0.433	31.41	Retain
S19	Cognitive control and adaptive reliance	0.517	0.701	0.498	34.61	Retain
S20	Cognitive control and adaptive reliance	0.291	0.540	0.420	42.94	Retain
S21	Evidence integration	0.210	0.479	0.429	43.08	Retain
S22	Cognitive control and adaptive reliance	0.619	0.791	0.566	27.18	Retain
S23	Evidence integration	0.645	0.735	0.530	31.51	Retain
S24	AI error detection	0.824	0.351	0.301	27.62	Retain

Note. Difficulty is the proportion of correct final decisions. Discrimination is the upper–lower 27% discrimination index.

**Table 9:** Fit Statistics for Competing Factor and Item Response Models.

Model	χ^2^/−2LL	df	CFI	TLI	RMSEA	SRMR	AIC	BIC	Model-Selection Conclusion
One-factor CFA	2106.06	252	0.799	0.780	0.097	0.063	—	—	Inadequate relative fit
Correlated multidimensional CFA	1207.78	249	0.896	0.885	0.070	0.043	—	—	Improved fit; CFI/TLI remain below preferred levels
Higher-order CFA	1207.78	249	0.896	0.885	0.070	0.043	—	—	Equivalent with three first-order factors; strong common component
Bifactor CFA	1118.39	228	0.904	0.883	0.071	0.043	—	—	Strong general factor; added complexity
Unidimensional 2PL	19,682.50	—	—	—	—	—	19,778.50	20,002.15	Inferior to multidimensional model
Multidimensional 2PL	19,454.97	—	—	—	—	—	19,556.97	19,794.60	Correlated 3D specification favored
Bifactor IRT	19,448.95	—	—	—	—	—	19,592.95	19,928.42	Added complexity not supported

Note. For IRT models, −2 log-likelihood is reported in the second column. AIC and BIC are reported for the full-information IRT models; they are not reported for the limited-information CFA models based on tetrachoric correlations.

**Table 10:** Multidimensional IRT Item Parameters and Measurement Information.

Item	Dimension	Discrimination (*a*)	Difficulty (*b*)	Infit	Maximum Information	*θ* at Maximum Information
S01	CAR	1.956	0.169	0.849	0.956	0.169
S02	CAR	1.789	0.625	0.861	0.800	0.625
S03	EI	1.578	−0.122	0.877	0.622	−0.122
S04	CAR	1.834	−0.429	0.875	0.841	−0.429
S05	ED	1.921	0.969	0.844	0.923	0.969
S06	ED	1.496	0.980	0.895	0.559	0.980
S07	ED	1.296	0.395	0.895	0.420	0.395
S08	EI	0.960	−0.958	0.944	0.231	−0.958
S09	CAR	1.364	1.479	0.924	0.465	1.479
S10	EI	1.150	0.399	0.924	0.330	0.399
S11	ED	1.761	−0.340	0.835	0.776	−0.340
S12	EI	1.357	0.054	0.899	0.460	0.054
S13	EI	1.737	0.858	0.865	0.754	0.858
S14	ED	2.126	−0.617	0.769	1.130	−0.617
S15	ED	0.889	−0.999	0.946	0.198	−0.999
S16	ED	1.973	1.144	0.850	0.973	1.144
S17	CAR	1.687	−1.159	0.898	0.712	−1.159
S18	EI	1.337	0.169	0.904	0.447	0.169
S19	CAR	1.761	−0.061	0.879	0.776	−0.061
S20	CAR	1.379	0.878	0.914	0.475	0.878
S21	EI	1.695	1.162	0.884	0.718	1.162
S22	CAR	2.497	−0.377	0.790	1.559	−0.377
S23	EI	2.094	−0.485	0.800	1.096	−0.485
S24	ED	1.133	−1.685	0.946	0.321	−1.685

Note. ED = AI error detection; EI = evidence integration; CAR = cognitive control and adaptive reliance.

**Table 11:** Joint Accuracy–Response-Time Model Estimates.

Parameter	Estimate	*SE*	95% CI	*p*	Interpretation
Cognitive accuracy–processing efficiency association	r = 0.249	—	[0.181, 0.315]	<.001	Higher accuracy accompanied greater processing efficiency
Item difficulty → log response-time intensity	0.646	0.095	[0.448, 0.844]	<.001	More difficult scenarios required longer processing
Correct response → log response time	−0.060	0.004	[−0.067, −0.053]	<.001	Correct decisions were approximately 5.8% faster
Highest-efficiency quartile vs. others: accuracy difference	0.092	—	—	<.001	No evidence of superficial fast responding

**Table 12:** Scenario-Level Associations With Final Decision Accuracy.

Predictor	*B*	Odds Ratio	*SE*	95% CI for OR	*p*
Correct AI advice	1.844	6.32	0.327	[3.22, 12.43]	<.001
High AI confidence	0.616	1.85	0.481	[0.69, 5.01]	.213
Time pressure	1.372	3.94	0.482	[1.46, 10.68]	.009
Interruption	0.046	1.05	0.216	[0.67, 1.64]	.833
High information conflict	−0.341	0.71	0.364	[0.33, 1.51]	.359
High task complexity	−0.043	0.96	0.312	[0.50, 1.83]	.892
ICAR-16	0.311	1.36	0.037	[1.26, 1.47]	<.001
Working memory	0.100	1.10	0.036	[1.03, 1.19]	.011
Processing speed	0.058	1.06	0.033	[0.99, 1.13]	.097
Attentional control	0.342	1.41	0.036	[1.31, 1.52]	<.001
AI literacy	0.155	1.17	0.035	[1.09, 1.26]	<.001
AI correctness × High confidence	−1.171	0.31	0.607	[0.09, 1.09]	.066
AI correctness × Time pressure	−3.090	0.046	0.491	[0.017, 0.126]	<.001
Interruption × Working memory	0.064	1.07	0.059	[0.94, 1.20]	.288

Note. Continuous person-level predictors were standardized. Standard errors account for clustering by both participant and scenario. Because the smaller clustering dimension contained 24 scenarios, reported confidence intervals and *p* values use a conservative t23 reference distribution. Scenario characteristics were fixed properties of the fractional design; coefficients therefore represent adjusted associations rather than isolated causal effects. The within-assessment 23-scenario rest score is not included in this primary model and is reported only in sensitivity analyses.

**Table 13:** Confidence Calibration Across AI-Advice Scenario Subsets.

AI-Advice Scenario Subset	Mean Accuracy	Mean Confidence	Calibration Bias	Absolute Calibration Error	Brier Score
Correct AI advice	0.526	0.632	0.105	0.400	0.198
Incorrect low-confidence AI advice	0.451	0.559	0.109	0.419	0.202
Incorrect high-confidence AI advice	0.404	0.628	0.224	0.434	0.230
Time-pressure scenarios	0.460	0.569	0.109	0.419	0.203
Overall	0.475	0.615	0.141	0.414	0.208

**Table 14:** Convergent, Discriminant, and Concurrent Criterion-Related Validity Results.

External Measure	Expected Relationship	Observed Association	95% CI	Validity Interpretation
Fluid/general reasoning ability (ICAR-16)	Moderate positive	r = 0.461 ***	[0.403, 0.514]	Moderate convergent evidence
Working memory	Moderate positive	r = 0.343 ***	[0.279, 0.403]	Convergent evidence
Processing speed	Small-to-moderate positive	r = 0.259 ***	[0.193, 0.324]	Convergent evidence
Attentional control	Moderate positive	r = 0.440 ***	[0.382, 0.495]	Convergent companion-measure evidence
AI literacy	Positive but weaker than cognitive measures	r = 0.246 ***	[0.179, 0.311]	Related but distinguishable
Trust in AI	Weak or negligible	r = −0.007	[−0.077, 0.063]	Negligible association; distinct from focal performance
AI self-efficacy	Small positive	r = 0.161 ***	[0.092, 0.229]	Related but clearly distinct
AI-use frequency	Weak positive	ρ = 0.063	[−0.008, 0.134]	Weak, nonsignificant association
Organizational decision quality	Moderate positive	r = 0.408 ***	[0.348, 0.465]	Concurrent criterion-related evidence

Note. *N* = 780. Pearson correlations are reported except for AI-use frequency, for which Spearman’s ρ was used because the measure was ordinal. *** *p* < 0.001.

**Table 15:** Hierarchical Regression of Concurrent Organizational Decision Quality.

Model	Predictors Added	*R* ^2^	Adjusted *R*^2^	Δ*R*^2^	10-Fold CV *R*^2^	CV RMSE	Model-Comparison Statistic
Model 1	Demographics	0.008	−0.002	0.008	−0.012	1.936	Overall F(8, 771) = 0.78, *p* = .623
Model 2	Work experience	0.008	−0.004	<0.001	−0.014	1.937	ΔF(1, 770) = 0.06, *p* = .808
Model 3	AI experience	0.015	0.001	0.007	−0.010	1.934	ΔF(2, 768) = 2.70, *p* = .068
Model 4	ICAR-16 + CRT + working memory + processing speed	0.090	0.072	0.075	0.059	1.867	ΔF(4, 764) = 15.68, *p* < .001
Model 5	AI literacy + trust + AI self-efficacy	0.093	0.072	0.003	0.055	1.871	ΔF(3, 761) = 0.92, *p* = .430
Model 6	Focal AI-advice assessment	0.196	0.176	0.103	0.160	1.764	ΔF(1, 760) = 97.04, *p* < .001

Note. Demographic controls included age, gender, education, and managerial status, with categorical predictors represented by the required indicator variables. AI experience included AI-use frequency and prior generative-AI experience (Limited, Moderate, Extensive). The conventional cognitive-performance block comprised ICAR-16, cognitive reflection, working memory, and processing speed. Attentional control was excluded from this primary block because its classification as a conventional cognitive measure was not established; a separate sensitivity analysis including attentional control is reported in the Supporting Information. Ten-fold cross-validation was used as an internal assessment of statistical stability and is not interpreted as prospective external validation.

**Table 16:** Measurement Invariance Results Across Employee Groups.

Group Comparison	Invariance Level	CFI	RMSEA	ΔCFI	ΔRMSEA	Conclusion
Gender	Configural	0.977	0.015	—	—	Supported
	Loading	0.974	0.015	−0.003	+0.001	Supported
	Threshold	0.975	0.015	+0.001	−0.001	Supported
Age	Configural	0.984	0.012	—	—	Supported
	Loading	0.978	0.014	−0.005	+0.002	Supported
	Threshold	0.978	0.014	0.000	0.000	Supported
Education	Configural	0.980	0.014	—	—	Supported
	Loading	0.981	0.013	+0.001	−0.001	Supported
	Threshold	0.984	0.012	+0.003	−0.001	Supported
Managerial status	Configural	0.992	0.009	—	—	Supported
	Loading	0.992	0.009	0.000	0.000	Supported
	Threshold	0.994	0.007	+0.002	−0.001	Supported
AI-use frequency	Configural	0.975	0.015	—	—	Supported
	Loading	0.976	0.015	+0.002	−0.001	Supported
	Threshold	0.975	0.015	−0.001	0.000	Supported
Occupational group	Configural	0.976	0.015	—	—	Supported
	Loading	0.974	0.015	−0.002	0.000	Supported
	Threshold	0.960	0.019	−0.014	+0.003	Full threshold invariance not supported

Note. Reference groups were male (*n* = 427), age < 40 years (*n* = 415), bachelor’s degree or below (*n* = 498), nonmanager (*n* = 546), daily-or-more AI use (*n* = 433), and operations-oriented occupations (*n* = 374). Comparison groups were female (*n* = 348), age ≥ 40 years (*n* = 365), postgraduate education (*n* = 282), manager (*n* = 234), weekly-or-less AI use (*n* = 347), and planning/analytical occupations (*n* = 406), respectively. The five participants identifying as nonbinary or another gender were retained in overall analyses but excluded from the two-group gender invariance analysis because of insufficient subgroup size. Residual invariance was not separately estimated for the dichotomous indicators.

**Table 17:** Differential Item Functioning Results Across Employee Groups.

Item Code	Group Comparison	Uniform DIF	Non-Uniform DIF	Effect Size Δ*R*^2^	Adjusted Probability Difference	Recommended Action
S19	Gender	Not significant, *q* = 0.701	Not significant, *q* = 0.150	0.009	+0.045	Retain
S24	Age	Not significant, *q* = 0.736	Not significant, *q* = 0.127	0.012	−0.020	Retain
S24	Education	Not significant, *q* = 0.851	Not significant, *q* = 0.887	0.004	+0.033	Retain
S16	Managerial status	Not significant, *q* = 0.511	Not significant, *q* = 0.844	0.007	−0.062	Retain
S01	AI-use frequency	Significant, *q* = 0.021	Not significant, *q* = 0.922	0.011	−0.101	Retain; monitor
S09	Occupational group	Not significant, *q* = 0.112	Not significant, *q* = 0.866	0.008	−0.063	Retain

Note. For comparisons without significant DIF, the item with the largest observed total DIF effect is shown. Benjamini–Hochberg correction was applied separately to uniform- and non-uniform-DIF test families within each group comparison. Adjusted probability differences are reported as comparison group minus reference group; negative values therefore indicate lower expected performance in the comparison group after matching on the remaining-item score. ΔMcFadden *R*^2^ values below approximately 0.02 were treated as small.

## Data Availability

The de-identified study dataset used to reproduce the reported analyses is provided as a supplementary data file accompanying the submission. The Supporting Information provides the scenario blueprint, scoring dictionary, analysis specifications, and sensitivity results needed to interpret and reproduce the reported analyses. The original contributions presented in this study are included in the article/Supplementary Materials. Further inquiries can be directed to the corresponding author.

## Supplementary Materials

The following supporting information can be downloaded at: https://www.mdpi.com/article/10.3390/jintelligence14090223/s1, The Supporting Information accompanying this article contains detailed methodological, psychometric, and reproducibility materials. Table S1, Complete Organizational Scenario Blueprint, presents the full scenario-level design and coding; Table S2, Full Expert Content-Validity Results, reports item-level expert evaluations; Table S3, Cognitive Interview Findings and Item Refinements, summarizes the cognitive-interview evidence; Table S4, Complete Pilot Item Statistics, reports the pilot results; and Table S5, Full Main-Sample Item Descriptive Statistics, reports item-level performance in the final analytic sample. Table S6, CFA and IRT Model Comparisons, presents the dimensionality results; Table S7, Multidimensional IRT Item Parameters, reports item-level parameters and information; Table S8, Response-Time Screening and Sensitivity Analyses, documents response-time diagnostics; Table S9, Primary Two-Way Cluster-Robust Logistic Model of Scenario-Level Decision Accuracy, reports the revised primary scenario-level model; Table S10, Measurement Invariance Results, reports the group-comparability analyses; Table S11, Differential Item Functioning Results, provides complete item-level DIF results; Table S12, Robustness and Internal Cross-Validation Analyses, reports sensitivity and internal cross-validation analyses; Table S13, Hypothesis-to-Test Alignment and Decision Summary, links each hypothesis to its hypothesis-aligned test and conclusion; and Table S14, Variable and Scoring Dictionary, defines the principal variables, coding rules, and scoring procedures. The Supporting Information also contains Figure S1, Item Characteristic Curves; Figure S2, Item Information Curves; Figure S3, Response-Time Distributions; Figure S4, Differential Item Functioning Plots; and Figure S5, Incremental Concurrent Criterion Validity and Internal Cross-Validation. Supplementary S1 provides the Full Assessment Instructions; Supplementary S2 contains the Complete 24 Organizational Scenarios and Scoring Key; Supplementary S3 provides the Expert Evaluation Form; Supplementary S4 contains the Cognitive Interview Protocol; Supplementary S5 presents the Analysis Workflow and Reproducibility Information; and Supplementary S6 contains the Questionnaire and Companion Measures used in the study.
